# FibriLysins disrupt *Staphylococcus aureus* biofilms and abscess communities

**DOI:** 10.1038/s41564-026-02513-1

**Published:** 2026-10-23

**Authors:** Vincent De Maesschalck, Marco Chittò, Claudia Siverino, Lena Gens, Stephan Zeiter, Alisa Hangartner, Sandrine Harzé, Eliane Vanhoffelen, Camille Van Laere, Alison Kerremans, Marleen Voet, Maarten Boon, Tine Van Win, Greetje Vande Velde, Jeroen Wagemans, Willem-Jan Metsemakers, Robert Geoffrey Richards, Thomas Fintan Moriarty, Rob Lavigne

**Affiliations:** 1 Laboratory of Gene Technology, Department of Biosystems, KU Leuven, Leuven, Belgium; 2 AO Research Institute Davos, Davos, Switzerland; 3 Biomedical MRI, Department of Imaging and Pathology, KU Leuven, Leuven, Belgium; 4 Department of Trauma Surgery, University Hospitals Leuven, Leuven, Belgium; 5 Department of Development and Regeneration, KU Leuven, Leuven, Belgium; 6 Center for Musculoskeletal Infection, University Hospital Basel, Basel, Switzerland

**Keywords:** Molecular medicine, Antibiotics

## Abstract

The rise of antibiotic resistance, particularly among pathogens such as methicillin-resistant *Staphylococcus aureus*, necessitates innovative antibacterial strategies. Here we introduce FibriLysins, a class of engineered enzymes combining staphylokinase and endolysin, which dismantle fibrin-encapsulated multicellular staphylococcal communities, including biofilms and staphylococcal abscess communities (SACs). We characterized diverse staphylokinases, developing a library of 27 FibriLysins, with a 78% positive hit rate. The lead variant, FL-028, has significant antibacterial activity against prevalent clinical *S. aureus* isolates. In vitro, FL-028 reduced bacterial counts in SACs by 3.4 log and in biofilms by 2.3 log. In vivo, FL-028 exhibits superior efficacy in a mouse model of bone fracture-related infection, outperforming vancomycin by 1 log and eliminating SAC presence in tissues. FL-028 shows no adverse effects on bone healing, as confirmed by histological examination, mechanical testing and micro-computed tomography analyses. These preclinical findings highlight the potential of FibriLysins as a robust and safe therapeutic approach against difficult-to-treat staphylococcal infections.

## Abstract (web summary)

Engineered FibriLysins dismantle fibrin-encapsulated *Staphylococcus aureus* communities and outperform vancomycin in a preclinical model of fracture-related infection.

## Main

Antibiotics have transformed human medicine by providing effective treatment for infectious diseases and dramatically reducing the risk of life‑threatening infections that once made surgery and other invasive procedures extremely hazardous. As a result of their impact and widespread use in these settings, resistance to antibiotics has surged. The majority of the antibiotics or their derivates used today have been discovered between 1940 and 1960^1^. The extensive scientific and financial investments required for the development of new antibacterials has led to a receding clinical pipeline contemporary to an antibacterial resistance crisis. Governmental bodies closely monitor this ‘silent pandemic’ and have established priority lists to focus drug development on the most urgent of pathogens^2–4^. In this regard, funding initiatives have been pulling innovations with different mode of actions to the market, resulting in a 136% increase of the number of phase 1 candidates from 2015 to 2022 (ref. ^5,6^).

In these priority lists, methicillin-resistant *Staphylococcus aureus* (MRSA) is consistently listed as a pathogen with high priority for developing new antibacterials, primarily due to its high burden to patients and the health care system in general^4^. It is estimated that half of the infections in high-income countries attributed to antimicrobial resistance involve either this pathogen or *Escherichia coli*^7^*. S. aureus* can cause a wide range of infections ranging from skin and soft tissue infections to blood stream infections to surgical site infections and medical device-related infections such as orthopaedic device-related infections^8^. Importantly, *S. aureus* is also a human skin commensal with 30% of the population being carriers^9^. As a result of co-evolution with their host, *S. aureus* encodes several human-specific virulence factors, but also various mechanisms enabling persistence inside the human host, including the formation of staphylococcal abscess communities (SACs) and biofilms^10,11^. For both SACs and biofilm, which are inherently antibiotic tolerant, the elaboration of polymeric fibrin from host fibrinogen forms a crucial component of the physical structure of these related, but distinct bacterial aggregates. These fibrin structures, known as pseudocapsules when surrounding SACS, limit immune cell infiltration and the diffusion of certain antibiotics^12–14^. Mechanistically, this pseudocapsule results from the conversion of host fibrinogen into fibrin, a key strategy exploited by *S. aureus* through virulence factors such as coagulase, the von Willebrand factor-binding protein and clumping factor A^15^. Because nutrients and oxygen are scarce in these environments, cells within a SAC are metabolically diverse^11^, which again reduces the efficacy of the limited amount of antibiotic able to penetrate the pseudocapsule^16^.

The physical separation of *S. aureus* aggregates provides protection to the bacterium in in vivo conditions, although there are also mechanisms for escape and dispersal, whereby shifts in virulence factor expression may be initiated, with a central role for the *S. aureus* accessory gene regulator (agr) system^17^. One of these agr-controlled virulence factors, prominent in *S. aureus* pathogenesis, is the prophage-encoded staphylokinase (SAK)^18^. This virulence factor stimulates the conversion of plasminogen into plasmin, which eventually results in fibrin degradation^19^, thereby facilitating the escape and spread of *S. aureus* into surrounding tissues^20^. Recombinant SAK was developed clinically as a fibrinolytic agent for patients with acute thrombotic cardiovascular conditions, while in the context of *S. aureus* infection, it is best known as a secreted virulence factor that modulates fibrin architecture and host interactions, rather than as a therapeutic antimicrobial^21^.

Bacteriophages and compounds derived from them represent an intensively studied sector in the (pre)clinical phase of antibacterial development^22^. In this field, a major role is played by bacteriophage-encoded peptidoglycan hydrolases or endolysins: enzymes degrading the peptidoglycan layer eventually leading to cell lysis. The strong antibacterial properties of endolysins, together with a generally accepted low chance of resistance development^23^, has earmarked them as a promising class of antibacterials^24^. Endolysins hold several advantages over conventional antibiotics as they are not dependent on the metabolism of the bacterium to be effective. As a result, several endolysins have been reported to have antipersister activity^25,26^, the potential to eradicate biofilms^25,27,28^ or small-colony variants^29^. However, continuous efforts are being performed to increase its clinical potential^30^.

Although bacteriophage-derived endolysins have been established as effective antibacterials against *S. aureus*, including its biofilm activity^31^, their combination with SAK or related fibrin-targeting strategies has not been described. The principal concept of our work is to utilize SAK to locally degrade the fibrin surrounding multicellular *S. aureus* aggregates and couple these with designer endolysins in so-called FibriLysins, effectively combining both enzymatic actions. To achieve this, we identified and characterized the known set of SAKs and validated their potential as dispersive agents of multicellular *S. aureus* communities. Based on key selection criteria, we next developed the FibriLysins and validated their activity in in vitro biofilm, SAC models and an in vivo model of *S. aureus* infection to assess in vivo efficacy and impact on bone healing as a safety outcome assessment.

## Results

### Development of FibriLysins

**Fig. 1:**
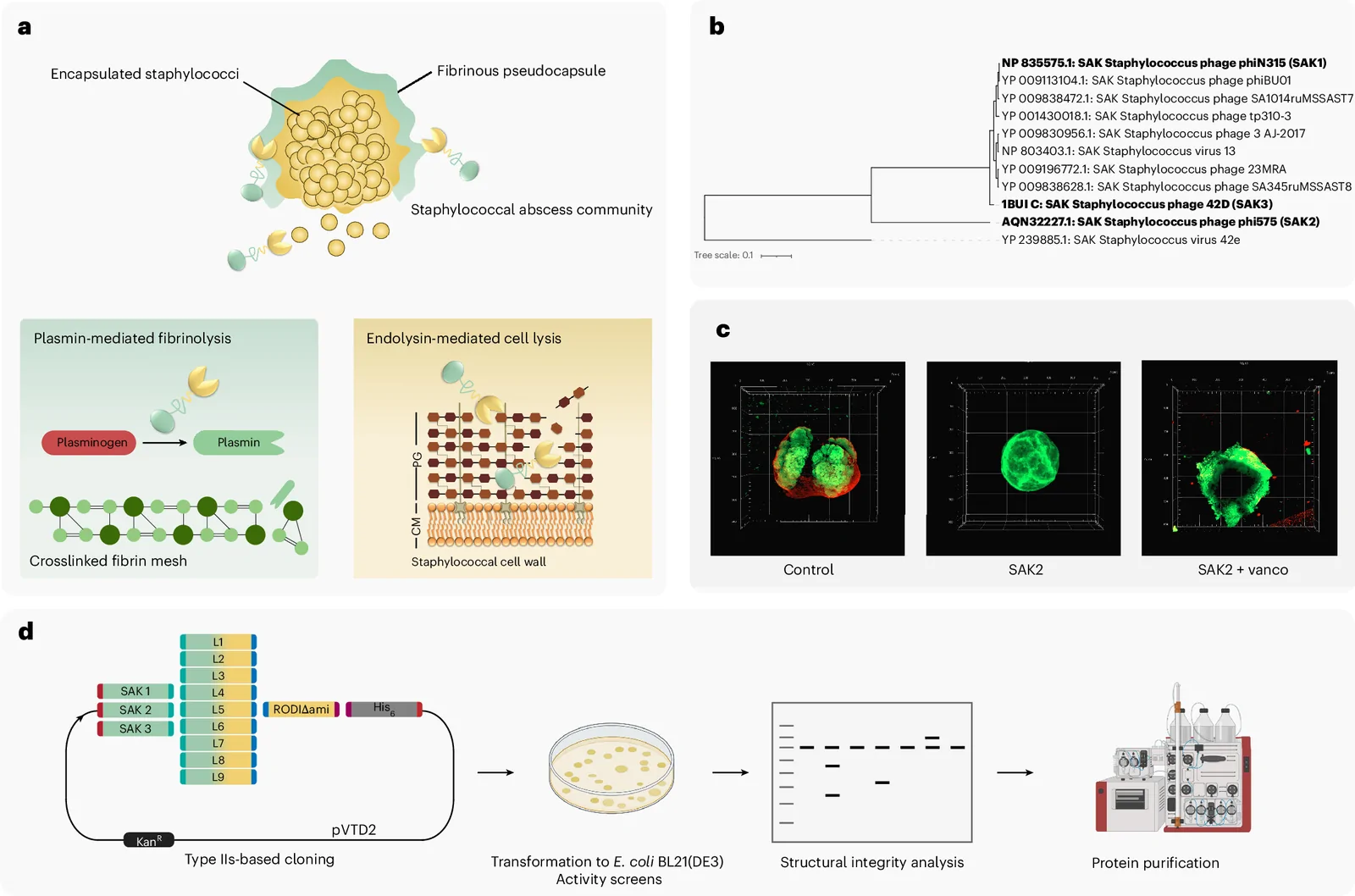
Schematic overview of the FibriLysin concept and its development process. **a**, Concept: bacteria reside in fibrin-embedded multicellular communities such as SACs and biofilms, limiting the efficacy of standard-of-care antibiotics. In a previously undescribed approach, we fused SAK as a plasminogen activator with endolysins, a potent class of antibacterials. This first module (SAK) stimulates fibrin degradation and hence provides an entry for the endolysin to the bacteria residing in multicellular communities (Extended Data Fig. 1). CM, cell membrane; PG, peptidoglycan. **b**, Phylogenetic tree of the publicly available SAKs identified in a sequence similarity search based on a known SAK (accession number P68802). This phylogenetic tree was based on a multiple sequence alignment using ClustalΩ^53,54^ and was visualized with iTOL^48^. Three diverse SAKs (highlighted in bold) were selected for further use in this work. **c**, Validation of SAK2 activity in our previously developed in vitro three-dimensional SAC model. Alexa Fluor 647 was used for the fibrin(ogen), while Syto9 dye was used to identify living bacterial cells. The red signal (fibrin) is completely absent when treated with SAK2 as a result of plasminogen activation. When combined with vancomycin (10 µg ml^−1^), SAC-residing cells were also killed, resulting in a decrease in green signal. **d**, Schematic overview of the proof-of-concept library created using the VersaTile platform^41^. The three selected SAKs were fused with nine different linkers to a previously characterized truncated endolysin with improved antistaphylococcal activity, RODI∆amidase^33^. These 27 different constructs were transformed to *E. coli* BL21(DE3) cells and screened for their muralytic activity. Following an SDS–PAGE/western-blot based structural integrity analysis, lead variants were purified using fast protein liquid chromatography (FPLC). Illustrations in **d** created in BioRender; Bens, L. https://biorender.com/he90fff (2026). Source data

The conceptual principle of a FibriLysin is to combine a plasminogen activator and a peptidoglycan-degrading moiety into a single designer enzyme. Because fibrin is present in multicellular staphylococcal communities, including SACs and biofilms^32^, this strategy targets a critical component contributing to bacterial persistence (Fig. 1a). In this manner, bacteria residing in these multicellular structures are no longer protected from the peptidoglycan-degrading moiety of the enzyme, in this case an endolysin. This, in turn, results in rapid cell lysis.

A sequence similarity search with SAK (accession number P68802) revealed the most diverse SAK variants available in public sequence databases (NCBI). Based on their protein sequences, a phylogenetic tree was established to select the three most diverse SAKs (Fig. 1b), termed SAK1-3 throughout this manuscript, for recombinant expression in *E. coli*. The plasminogen-activating properties of all three were validated in vitro (Fig. 1c and Extended Data Fig. 2). Next, a proof-of-concept library (Fig. 1d) was made, fusing these SAKs to a known endolysin (RODI∆ami) with potent antistaphylococcal activity^33^. The RODI∆ami is an engineered derivative of the *S. aureus* phage endolysin LysRODI, classified as a modular Gram‑positive endolysin composed of an N‑terminal N‑acetylmuramoyl‑L‑alanine amidase catalytic domain and a C‑terminal SH3b‑type cell‑wall binding domain. In its optimized form, the amidase domain was truncated to enhance catalytic efficiency and stability, yielding the high‑activity variant RODI∆ami used in this study. RODI∆ami was selected as the preferred endolysin as it emerged as the lead candidate from a panel of 12 engineered antistaphylococcal endolysins previously generated by modular domain shuffling, owing to its superior specific lytic activity, low minimal inhibitory concentration (MIC) values, strong biofilm‑removal ability and robust physicochemical stability^34^. The VersaTile platform subsequently enabled efficient assembly of the different fusion constructs. VersaTile is a modular DNA assembly framework that enables rapid, one-pot construction of combinatorial protein libraries by recombining predefined functional domains into a single open reading frame using a type IIS restriction enzyme-based strategy. Individual domains (‘tiles’) are stored with position-specific overhangs, allowing controlled assembly of fusion constructs with defined domain order and orientation. This approach facilitates efficient generation and parallel screening of diverse engineered lysin variants, supporting systematic exploration of structure–function relationships in a high-throughput manner (Extended Data Table 1). Following library construction, a first screening round was performed using crude lysates of the resultant fusion constructs from recombinant *E. coli* strains. Endolysin functionality was assessed using a muralytic assay on *S. aureus* peptidoglycan, while SAK activity was independently evaluated using a plasminogen activation assay. This initial functional screening yielded a positive hit rate of 78% (21 out of 27 constructs), indicating successful preservation of activity for both enzymatic components (Extended Data Fig. 3). From these hits, a subset of variants that showed robust activity and a clear dose-dependent response in the plasminogen activation assay was selected for further evaluation. These candidates were subsequently assessed for structural integrity by western blot analysis (Extended Data Fig. 3) and for antibacterial activity against a panel of five staphylococcal strains (four *S. aureus* strains and one *S. epidermidis* strain) following protein purification (Supplementary Table 2). Based on combined performance across these secondary validation criteria, the variant FL-028 was selected as the lead FibriLysin construct for subsequent in-depth characterization and in vivo studies.

FibriLysin FL-028 was then tested against an extended panel of 34 diverse clinical *S. aureus* isolates reflecting the diversity of both community-acquired as well as hospital-acquired isolates. FL-028 showed antibacterial activity against 30 of these isolates in a MIC assay (Extended Data Table 2). A phylogenetic tree based on the core genome of the tested isolates revealed that strains from diverse common clonal complexes were susceptible to FL-028 (Extended Data Fig. 4), and the four less susceptible isolates were also distributed across the tree. Furthermore, FL-028 was shown to retain its plasminogen-activating activity in heat-inactivated human serum, across a broad pH range and at elevated temperature (Extended Data Fig. 5). Finally, antibacterial activity of FL-028 was retained despite an increase in pooled human serum and plasma, indicating lack of any impact of potential antibodies retained by humans against SAK2 (Extended Data Fig. 7). Taken together, these data highlight the suitability of FL-028 for antibacterial testing in advanced, multicellular in vitro models.

### FibriLysins tackle SACs and biofilms in vitro

**Fig. 2:**
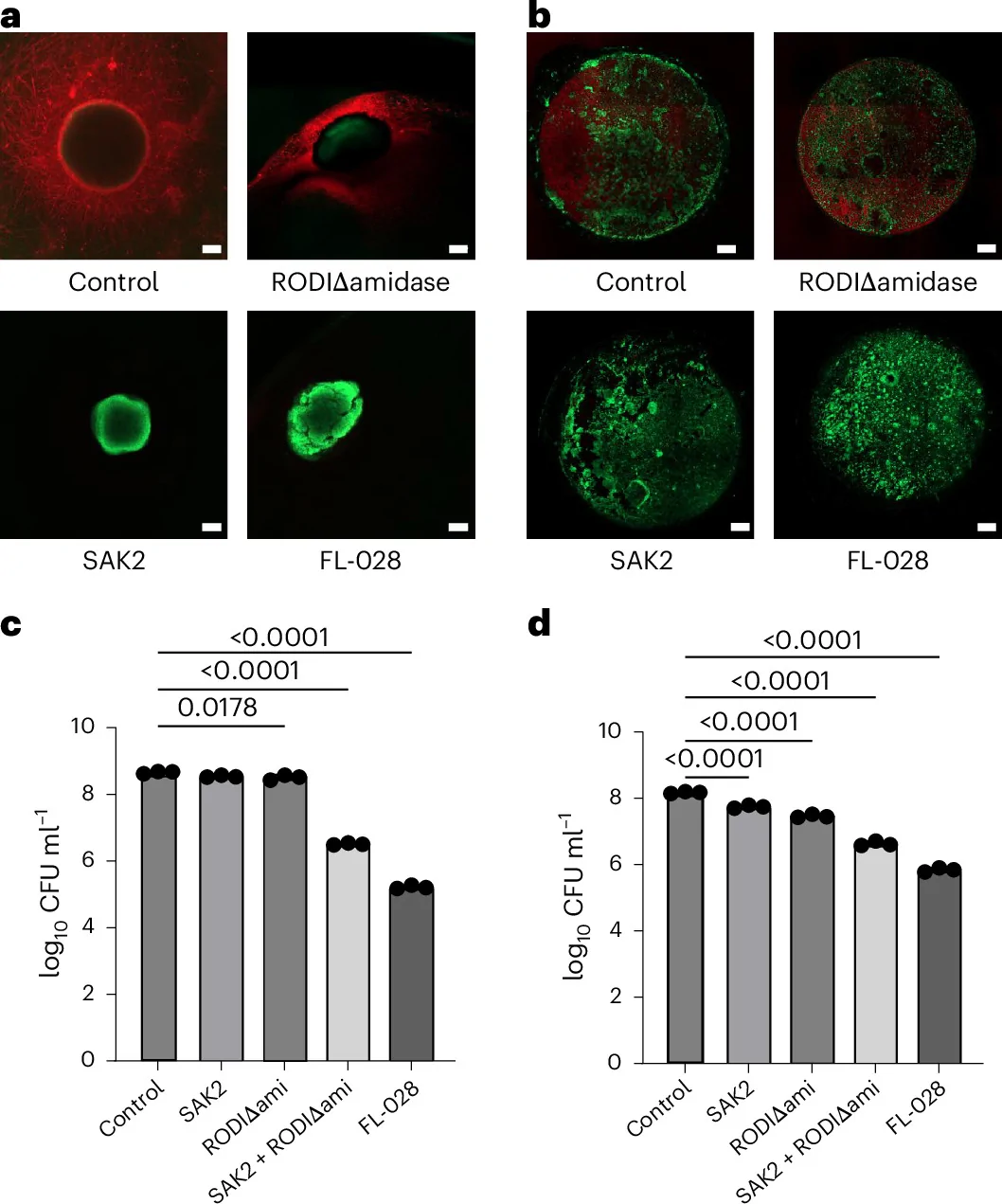
FibriLysins tackle SACs and biofilms in vitro. **a**, Representative CLSM images of SAC samples treated with PBS, RODI∆ami, SAK2 and FL-028. Alexa Fluor 647 was used to stain fibrin(ogen), while Syto9 dye was used to identify live bacterial cells. The fibrin signal was absent in the samples treated with SAK2 and FL-028. Scale bar, 100 µm. Representative images from one of three independent biological experiments are shown. **b**, Forty-eight-hour old biofilms were treated with FL-028 over a 2-day period, with a concentration of 10 µM per dose. Representative CLSM images of biofilm samples treated with the indicated treatment. Alexa Fluor 647 was used for the fibrin(ogen), while Syto9 dye was used to identify the live bacterial cells. Scale bar, 100 µm. Representative images from one of three independent biological experiments are shown. **c**, Treatment of mature SACs with FL-028 leads to a significant, 3.4 log_10_ reduction in bacterial counts (*P* = 0.0040). The fusion protein eradicates more bacteria compared with the fusion partners being applied as a mixture of individual components in a combined treatment (*P* = 0.0001). **d**, In the biofilm model, FL-028 treatment resulted in a 2 log_10_ reduction in CFU compared with the control group and a 1 log_10_ reduction compared to treatment with individual enzymes. All experiments were performed using *n* = 3 independent biological replicates, each analysed in triplicate technical replicates. Error bars represent one standard deviation from the mean of biological replicates. Normality of the data was calculated using the Shapiro–Wilk test (*P* < 0.05), after which significant differences between groups (*P* < 0.05) were shown using a two-sided one-way analysis of variance (ANOVA) of log_10_-transformed data and a Tukey’s multiple-comparisons test. Source data

FL-028 was first assessed in our previously developed in vitro three-dimensional SAC model^13^ in which *S. aureus* forms structured multicellular communities within a human plasma-supplemented collagen matrix and is surrounded by a fibrin-rich pseudocapsule. Upon maturation, these SACs were treated for 48 h with either 10 µM FL-028 or a mixture of its individual components (Fig. 2a). When these SACs are visualized with confocal laser scanning microscopy (CLSM) using specific staining for fibrin (Alexa Fluor 647, red) and living bacteria (Syto9, green), it is clear that no fibrin (red) signal could be observed in the SACs treated with either SAK2 or FL-028. Fibrin could still be observed in the control sample or when the endolysin RODI∆ami was used individually. In terms of antibacterial activity, the combination of SAK2 and RODI∆ami is required to significantly reduce the number of bacteria (Fig. 2c). Importantly, treatment with the FibriLysin resulted in a reduction of 3.4 log_10_ units, whereas the combination of both individual proteins only reduced bacterial counts by 2.1 log_10_ units, implying a significant benefit in combining both into one single molecule (*P* = 0.0001).

Subsequently, the performance of FL-028 was evaluated against biofilms (Fig. 2b). *S. aureus* biofilms were grown for 48 h on titanium disks in tryptic soy broth (TSB) supplemented with 1% human plasma. Similar to observations in the SAC model, no fibrin could be detected during CLSM after SAK2 or FL-028 treatment, while a clear red signal was obtained for the untreated control and the endolysin-only-treated sample, indicating the presence of fibrin (Fig. 2b). Furthermore, FL-028 treatment resulted in a significant 2.3 log_10_ reduction in colony-forming units (CFU) compared with the untreated control (Fig. 2d). Moreover, a significant difference was observed between FL-028 and the unfused moieties of this fusion protein, suggesting that, once again, both fusion partners benefit from spatial proximity. Activity was also confirmed against *S. epidermidis* biofilms (Extended Data Fig. 7a), which do not incorporate fibrin, indicating that antibacterial activity extends beyond *S. aureus* biofilms, and in the absence of fibrin.

**Fig. 3:**
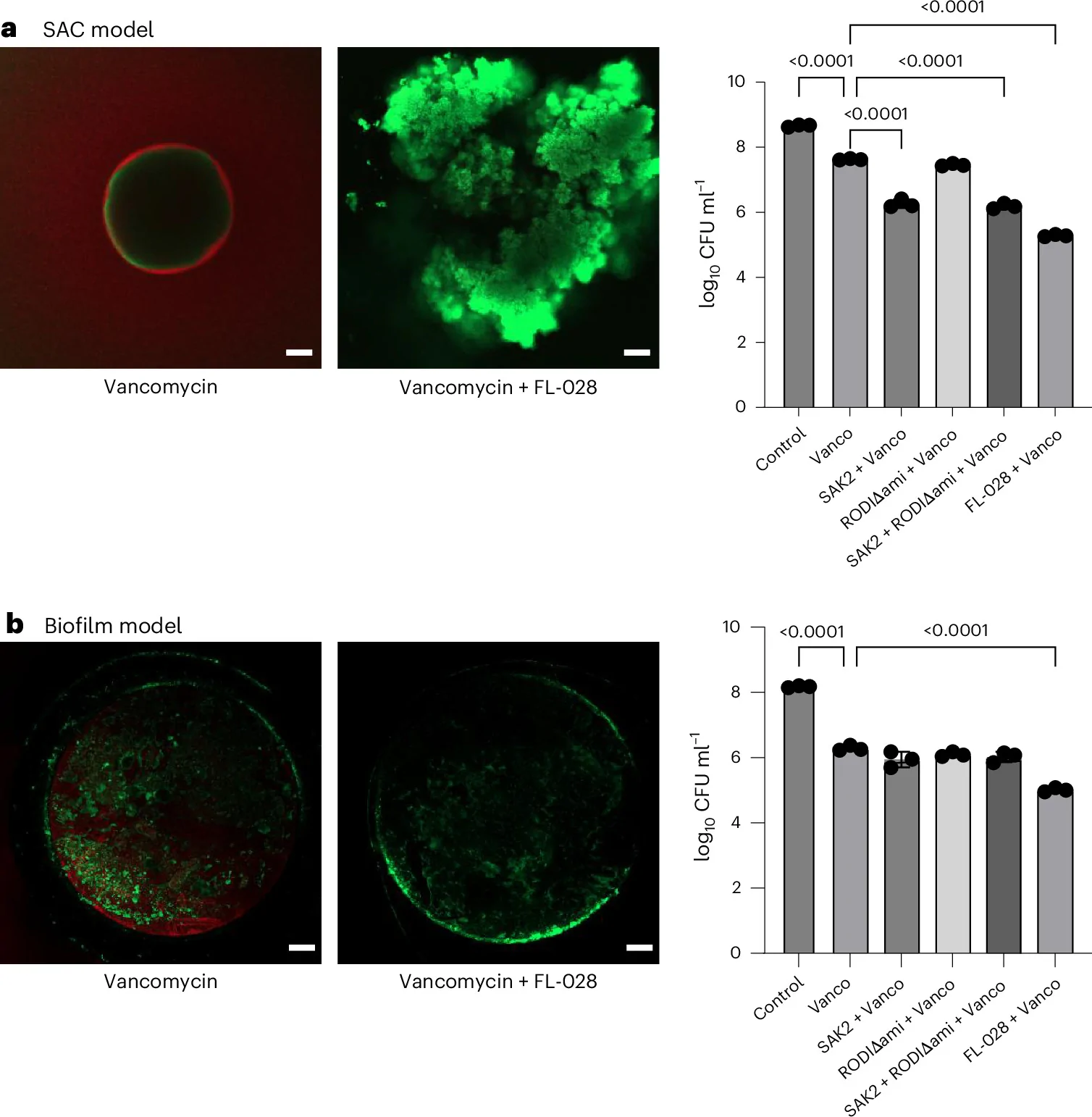
FibriLysin FL-028 outperforms the standard-of-care antibiotic vancomycin in SAC and biofilm models. **a**, Combination of the standard of care, vancomycin with FibriLysin FL-028, or its components in the in vitro SAC model. SAK2 potentiates vancomycin by providing access to bacterial communities inside the SAC. A combination treatment results in a reduction of the number of bacterial counts. All experiments were performed using *n* = 3 independent biological replicates, each analysed in triplicate technical replicates. Error bars represent one standard deviation from the mean of biological replicates. Representative CLSM images of SAC samples treated with the indicated treatment are shown. Scale bar, 100 µm. **b**, Combination treatments with the standard-of-care vancomycin and indicated superiority when combined with FL-028 in the biofilm model. FL-028 showed a 3 log_10_ reduction when combined with vancomycin. All experiments were performed using *n* = 3 independent biological replicates, each analysed in triplicate technical replicates. Error bars represent one standard deviation from the mean of biological replicates. Representative CLSM images of biofilm disks samples treated with the indicated treatment are shown, with red colour indicating fibrin. Scale bar, 500 µm. Normality of the data was calculated using the Shapiro–Wilk test (*P* < 0.05), while significant differences between vancomycin treatment and other groups (*P* < 0.05) were shown using a two-sided one-way ANOVA of log-transformed data and a Tukey’s multiple-comparisons test. Source data

The activity of SAK2, RODI∆ami and FL-028 was also evaluated in combination with the standard-of-care vancomycin in the SAC model. Interestingly, the standard-of-care antibiotic alone was barely able to kill any of the bacteria residing in the SAC (Fig. 3a) (10 µg ml^−1^, 10 × MIC), whereas it could exert its antibacterial effect when combined with SAK2. As such, we can state that the pseudocapsule inhibits vancomycin activity and SAK2 potentiates it in this model. The combination of vancomycin with FL-028 resulted in a significant reduction in CFU compared with both the control (*P* = 0.0029) and vancomycin alone (*P* = 0.0294). In short, FL-028 outperforms the standard-of-care antibiotic, vancomycin, in this model, whereas a combination treatment results in further reduction of the bacterial burden.

In contrast to observations in the SAC model, a SAK2 treatment in combination with vancomycin did not result in further reduction of bacterial burden in the biofilm model (Fig. 3b). However, when vancomycin was combined with FL-028, bacterial burden was reduced significantly further, resulting in a 3.2 log_10_ killing of the biofilm population.

### Safety assessment of FibriLysin FL-028 in vitro and in vivo

**Fig. 4:**
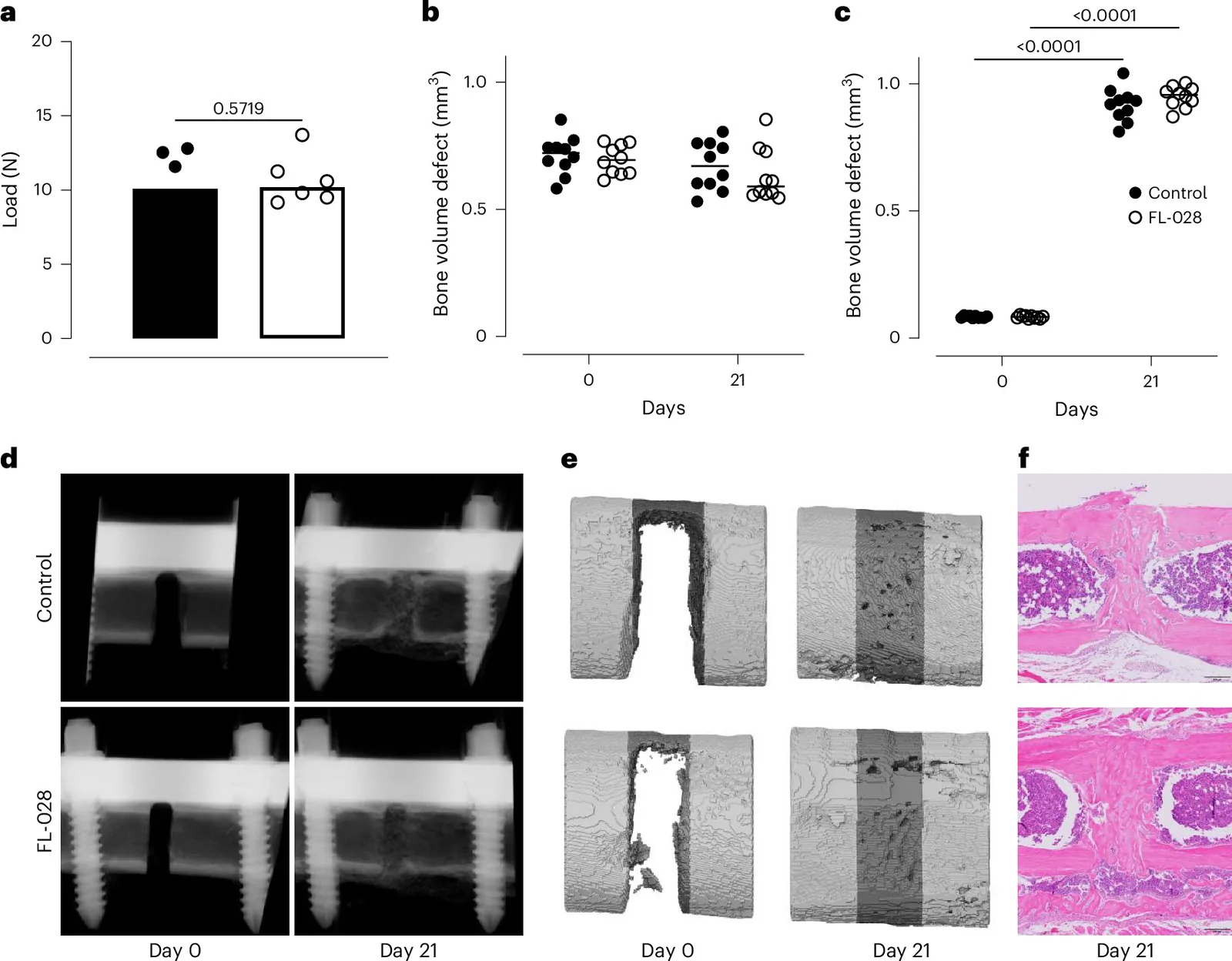
FL-028 does not negatively affect bone healing in a mouse fracture model. Safety aspects of the use of FL-028 was assessed in vivo in a mouse model with femoral osteotomy and plate fixation. **a**, Mechanical strength was assessed using a four-point bending test. Each data point represents one animal (biological replicate; *n* = 6 mice per group). Error bars indicate one standard deviation from the mean. Statistical comparisons were performed using a two-sided unpaired *t*-test (*t* = 0.5844, d.f. 10, *P* = 0.5719). **b**, No significant differences in bone volume could be observed between saline-treated mice (control) and FL-028, calculated by µCT. Each data point represents one animal (biological replicate; *n* = 10 mice per group). Error bars indicate one standard deviation from the mean. **c**, The bone volume in the defect was measured at day 0 and day 21. Each data point represents one animal (biological replicate; *n* = 10 mice per group). Both groups showed a similar increase in bone formation within the osteotomy site, resulting in comparable values at day 21. Error bars indicate one standard deviation from the mean. Statistical analysis was performed using a two-sided repeated measures two-way ANOVA. A significant effect of time was observed (*F*(1,9) = 3,923, *P* < 0.0001), whereas no significant effect of treatment (*F*(1,9) = 1.990, *P* = 0.1920) or interaction between time and treatment (*F*(1,9) = 2.297, *P* = 0.1639) was detected. **d**,**e**, µCT radiographs (**d**) and µCT reconstructions (**e**) are shown. **f**, Representative images from H&E staining both show the change over time (day 0 to day 21) with substantial bone formation observed in both groups. Scale bar, 200 µm. Source data

After confirming the potential of this class of engineered endolysin in vitro, its safety profile was assessed both in vitro and in vivo. No toxicity was observed against fibroblasts (Extended Data Fig. 7b). Following these satisfactory safety assessment, FL-028 was tested in a mouse model of bone fracture fixation. First, the fibrinolytic activity of FL-028 was evaluated in non-infected animals because delayed bone healing might be a concern when plasminogen activators are used as a therapeutic drug, considering the importance of fibrin to early stages of bone fracture healing. In this regard, SAKs display favourable properties as plasminogen activators because their interaction with plasminogen is rapidly inhibited by host factors such as alpha-2-antiplasmin (α_2_AP)^35^, therefore only stimulating local fibrinolysis. In this bone healing study, FL-028 (10 µM) or saline was administered locally at the osteotomy site by 50-µl injections once daily from day 5 to day 18, with *n* = 10 mice per group. Histological examination revealed no significant differences in bone bridging between the FL-028-treated group and untreated controls (Fig. 4). Moreover, mechanical testing further confirmed the structural integrity of the bone following FL-028 treatment (*P* = 0.5719). In addition, micro-computed tomography (µCT) analyses revealed no significant differences in bone volume between the two selected groups. As expected, we could also observe similar values of bone volume defect in both groups at day 21 of the trial (Fig. 4), suggesting active new bone formation that ultimately contributed to the bridging of the osteotomy site. We could therefore conclude that FL-028 can be considered as a safe molecule in this animal model.

### FL-028 outperforms vancomycin in an in vivo fracture-related infection model

**Fig. 5:**
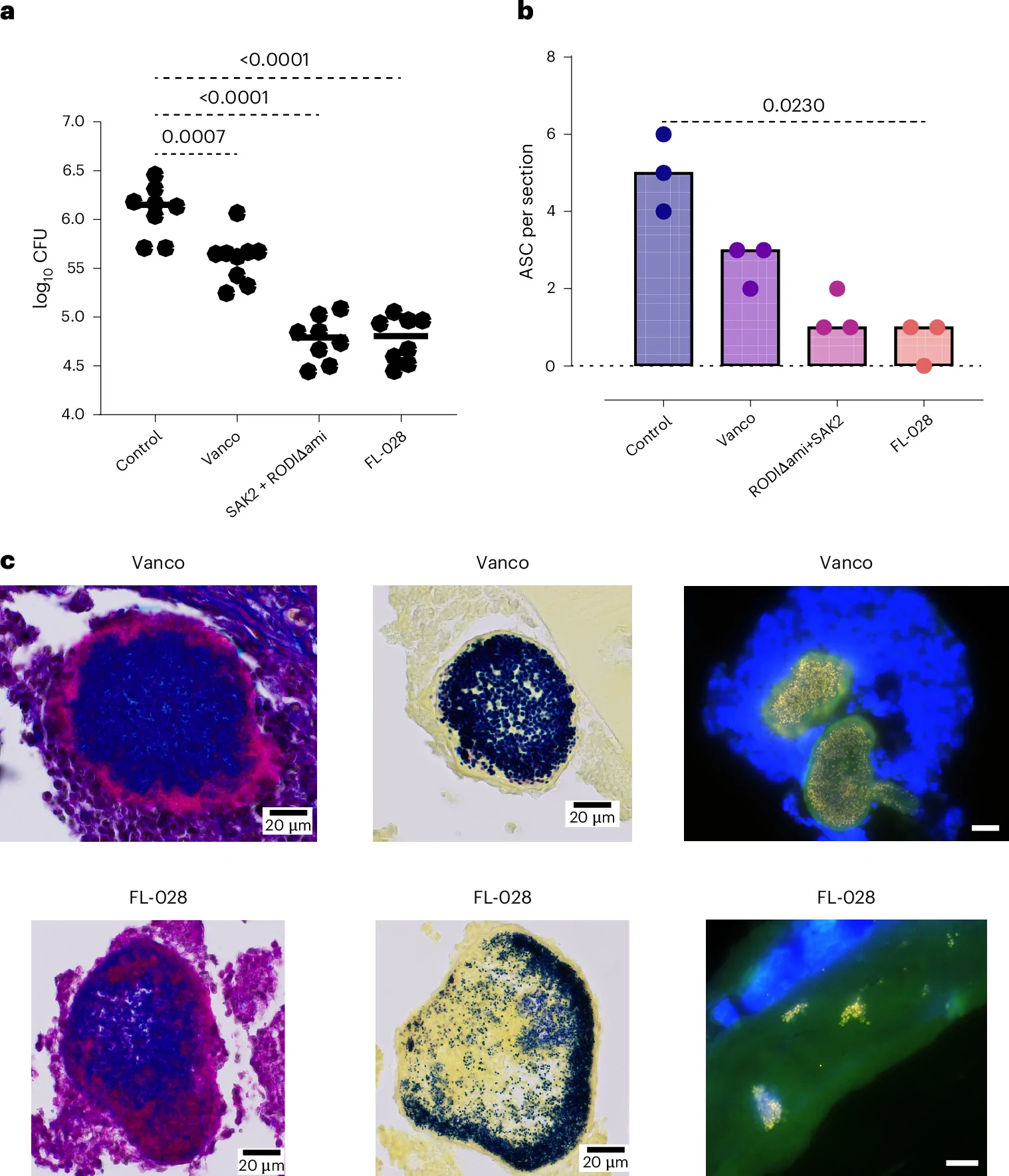
FL-028 outperforms vancomycin in an in vivo fracture-related infection model. **a**, After a 14-day treatment regimen (daily injections of 50 µl volume with the concentration of 10 µM FL-028 or both unfused enzymes locally at the osteotomy or subcutaneous injections of 2.75 mg vancomycin), animals were euthanized and the bacterial burden inside the bone was determined. Treatment with FL-028 or both unfused enzymes resulted in one further log reduction compared with the standard-of-care vancomycin. Statistical analysis was performed using a two-sided one-way ANOVA, followed by Tukey’s multiple-comparisons test. **b**, Superiority of this approach was also reflected in the number of staphylococcal abscess communities (SACs) that could be obtained from a tissue section, where this approach resulted in nearly zero retrieved SACs. **c**, Left: Picro-Mallory staining of tissue sections indeed confirms the hypothesized mode of action resulting in the degradation of fibrin in FL-028-treated sections compared with vancomycin-treated mice. Centre: Brown–Brenn (B&B) staining, a technique used to identify bacteria in tissue samples, also shows loss of integrity of the SAC structure in FL-028-treated mice. Right: FISH imaging confirms the absence of multicellular structures in FL-028-treated samples (scale bar 10 µm), which are present in vancomycin-treated samples (scale bar 20 µm). Each data point in **a** represents one animal (biological replicate; *n* = 8 mice per group). For histological analyses (**b** and **c**), *n* = 2–4 mice per group were evaluated. Source data

Next, our mouse fracture-related infection model was used to compare FL-028 with the standard of care, vancomycin (Extended Data Fig. 7c). After inducing *S. aureus* infection in a plate-stabilized osteotomy of the femur, the infection was surgically treated after 5 days and treatment with either FL-028 or vancomycin was started. FL-028 was administered once daily as a 50-µl local injection at the osteotomy site (10 µM) from day 5 to day 18, with *n* = 8 mice per group for bacteriological analyses and *n* = 2–4 for histology. The animals received a single daily injection FL-028 or both unfused enzymes locally at the osteotomy or subcutaneous injections of 2.75 mg vancomycin, or the combination of FibriLysin and vancomycin at the same doses. The bacterial burden in the bone tissue was determined after 14 days of treatment. Both FL-028 and the individual enzymes combined reduced bacterial burden in the bone significantly, outperforming the standard-of-care vancomycin by one log unit (Fig. 5). Combination of FL-028 and vancomycin did not further improve outcome compared with FL-028 alone (Extended Data Fig. 7c). Moreover, post-mortem histological analysis of stained sections confirmed superiority of the FibriLysin approach as significantly more multicellular communities were identified in the control and vancomycin-treated groups compared with the other groups where a fibrinolytic agent in combination with an endolysin was used, either fused or co-administered. The proposed mode of action could be validated as a reduction of the fibrous pseudocapsule surrounding the SACs was observed in Picro-Mallory- and Brown–Brenn-stained tissue sections when treated with FL-028 or a combination of both fusion partners (Fig. 5b,c). These findings were supported by fluorescence in situ hybridization (FISH), demonstrating the presence of defined bacterial aggregates in the control group, whereas the FL-028-treated group exhibited a more dispersed bacterial distribution. Overall, this provides histological evidence that FL-028 treatment indeed leads to substantial degradation of multicellular staphylococcal communities.

## Discussion

The antimicrobial resistance crisis represents a critical challenge in modern medicine, leading to a situation where standard treatments become increasingly ineffective, and the infections persist. Therefore, it is crucial to expand the antibacterial armamentarium beyond the traditional discovery platforms. While endolysins have been widely recognized as a promising class of antibacterials^24^, previous clinical trials with first-generation (natural phage enzymes used in their native form) and second-generation (engineering to optimize their catalytic and cell-wall binding domains) endolysins have struggled to demonstrate clear clinical superiority. This research aims to unlock their therapeutic potential by addressing limitations observed in earlier studies. One promising strategy is to incorporate key aspects of human pathogenesis into the design and application of these agents as a third-generation endolysin^30^. In particular, *S. aureus* infections are characterized by fibrin-embedded multicellular communities limiting diffusion of small-molecule antibiotics to metabolically diverse *S. aureus* cells. The FibriLysin concept emerged from the strategic application of SAK to dismantle the protective pseudocapsule at the right time and location, combined with an endolysin that remains active regardless of the bacterium’s metabolic state.

In this work, we demonstrate the potential of this approach, starting from a proof-of-concept library to hit-to-lead development with FL-028 and validation in vivo.

However, little is known about the biological role of SAK and its therapeutic (safety) profile. As SAK is a plasminogen activator, uncontrolled use might lead to negative impact on fracture healing. In this study, we provide both in vitro and in vivo evidence that locally applied FibriLysins do not cause adverse effects, specifically cytotoxicity to fibroblasts in vitro or impaired bone healing in mice in vivo. Although one of the limitations of endolysin therapy in general relates to circulating half-life^36^, the half-life of FibriLysins is less critical in the context of direct local injection, as performed in this study. Nevertheless, the optimal dosing regimen and route of administration remain to be determined before potential clinical translation^37^.

The selective pressure associated with the widespread use of antibiotics and thereby selecting for antibiotic-resistant strains is one of the reasons for the current antimicrobial resistance crisis. Therefore, it is important to develop evolutionary robust antibacterial strategies. Coagulation is a central and conserved feature of *S. aureus* pathogenicity, enabling the formation of fibrin‑based multicellular communities that protect the bacteria across diverse infection types. Because these protective structures are composed of host‑derived fibrin rather than bacterially encoded factors, there is no simple pathway for *S. aureus* to evolve resistance to fibrinolytic mechanisms that act directly on this fibrin scaffold. By focusing on a host‑derived virulence barrier that the pathogen cannot readily modify, fibrinolytic approaches such as FibriLysins provide an evolutionarily robust means of disrupting *S. aureus* multicellular communities. In addition, the endolysin moiety is also considered evolutionary robust as several studies fail to evoke resistance following exposure to subinhibitory concentrations.

A similar reasoning could be followed for combining plasminogen activators such as SAK with standard-of-care antibiotics. Indeed, upon fibrin degradation, embedded bacteria will be exposed to higher doses of antibiotics as diffusion is less limited. This increases the efficacy of existing treatments and reduces the likelihood of antibiotic resistance development given the reduced chance of bacteria to get exposed to subinhibitory concentrations. This highlights the potential of this first-in-class approach as a tool to tackle difficult-to-treat infections.

## Methods

### Molecular cloning

As SAK is an extracellular protein, the consensus cleavage sites of the respective signal sequences were predicted using DeepSig^38^, SignalP5.0^39^ and Predisi^40^. Next, the encoding genes were codon optimized for expression in *E. coli* and synthesized (IDT), readily containing the required restriction sites and position markers for VersaTile cloning^41^. These genes were cloned for later use on position 1 and position 3 into the entry vector pVTE^41^. In brief, genes were directly ligated into the *sapI* site of pVTE using a reaction mixture of 20 µl comprising 100 ng of pVTE, 50 ng of the sequence of interest, 2 µl of T4 DNA ligase buffer (Thermo Fisher Scientific), 3 U of T4 DNA ligase and 10 U of SapI. Chemocompetent *E. coli* TOP10 were transformed with 5 µl of this mixture and plated on lysogeny broth (LB) containing 1.5% agar, 5% sucrose and 100 µg ml^−1^ ampicillin allowing selection for cells containing a ligated plasmid devoid of *sacB*. Correct cloning was validated with Sanger sequencing (Eurofins Genomics, Germany). To create expression constructs, 50 ng of all selected pVTE plasmids in a single 20-µl mixture containing 100 ng pVTD2, 10 U BsaI, 3 U T4 DNA ligase and 2 µl T4 DNA ligase buffer. For both reactions, a cyclic restriction-ligation protocol was used^41^. Initially, the SAK of *Staphylococcus* virus 42e was included to cover complete genetic diversity of the publicly known SAKs (Fig. 1b) but failed to clone in the entry vector. Newly generated constructs are available from the corresponding authors upon reasonable request.

### Recombinant protein expression and purification

Multiple colonies of freshly transformed *E. coli* BL21(DE3) or ClearColi(DE3) were selected to inoculate an overnight culture, which constituted to 1.5% and 4.5% of the final expression volumes respectively. Cells were grown to an optical density at 600 nm (OD_600_) of 0.6 and induced with 1 mM isopropyl β-D-1-thiogalactopyranoside (Sigma-Aldrich). Depending on the protein encoded on the expression plasmid, optimal expression conditions differed (Table 1). Cells were collected by centrifugation and resuspended in lysis buffer (20 mM NaH_2_PO_4_, 500 mM NaCl, pH 7.4) containing 20 mM imidazole (Acros Organics) and 1 mM Pefabloc protease inhibitor (Merck). Following three freeze–thaw cycles at −80 °C, cells were sonicated and treated with 10 U DNase I (Thermo Fisher Scientific). Finally, the lysate was centrifuged (60,628*g*, 10 min, 4 °C) and filtered (0.22 µm).

**Table 1:** Expression conditions used for the recombinant proteins used in this work

Expression conditions	Used for
18 h, 16 °C	LysRODIΔami, SAK2-L1, SAK2-L2, SAK2-L3, SAK2-L4, SAK2-L5, SAK2-L6, SAK2-L7, SAK2-L8, SAK2-L9
18 h, 30 °C	SAK2, SAK3
4 h, 37 °C	SAK1

Next, proteins were purified using fast protein liquid chromatography using an AKTApure system (Cytiva). Fractions of interest were pooled and concentrated using Microsep Advanced Centrifugal Devices (3 kDa or 10 kDa MWCO, Pall Corporation), followed by dialysis to storage buffer (20 mM NaH2PO4, 150 mM NaCl, 40 mM L-histidine, 40 mM L-arginine, pH 7.4) unless mentioned otherwise. Protein concentration was determined by measuring absorbance at 280 nm (SimpliNano) or using the Qubit protein BR assay kit (Invitrogen). Samples were analysed using a 12% (FibriLysins) or a 16% (SAKs) SDS–PAGE gel.

### Halo-based muralytic assay

*E. coli* BL21(DE3) transformants were screened for muralytic activity of their recombinantly expressed FibriLysin by streaking on LB containing 2% agar, 0.05 mM isopropyl β-D-1-thiogalactopyranoside, 50 µg ml^−1^ kanamycin, 5% sucrose and 1% *S. aureus* cell wall substrate. The latter was prepared by autoclaving a 300-ml culture, followed by centrifugation (4,000*g*, 30 min, 4 °C). The pellet was washed twice in 20 ml phosphate-buffered saline (PBS). After the last round of centrifugation, the pellet was resuspended to a suspension to 0.2 g cells ml^−1^ PBS. Plates were grown overnight at 30 °C followed by 3–7 days of incubation at room temperature. The formation of a translucent halo surrounding a colony indicates muralytic activity of the overexpressed FibriLysin.

### Plasminogen activation assay

Protein samples were mixed with 0.01 units ml^−1^ plasminogen (Sigma-Aldrich) and 1 mM D-Val-Leu-Lys-4-nitroanilide dihydrochloride (Sigma-Aldrich) in a 10-µl volume. The samples were incubated at 37 °C while absorbance at 405 nm was measured every 3 min using a Clariostar Plus (BMG Labtech) multimode plate reader.

### MIC assay

To assess the antibacterial potential of recombinant proteins, a MIC assay was performed. In brief, 10^6^ bacteria were incubated with a twofold dilution of the tested protein in 200 µl Mueller–Hinton broth (BD). Following overnight incubation at 37 °C, the MIC was determined by interpreting the well with the lowest protein concentration where growth inhibition took place.

### Genome analysis of tested clinical isolates

Genomes of the tested strains that were not publicly available were sequenced in house using specific sequencing methods. Genomic DNA was isolated using the Genejet genomic DNA purification kit (Thermo) according to the manufacturer’s instructions. Genomes were sequenced using either Illumina (Illumina MiniSeq) or Oxford Nanopore (MinION or PromethION) sequencing platforms or a combination of both (Supplementary Table 1). Fastq files were trimmed using Trimmomatic^42^ (v.039) or Porechop (v0.2.4, https://github.com/rrwick/Porechop) for Illumina and Nanopore data respectively. Next, genomes were assembled de novo using Unicycler^43^ (v0.5.1). Assembled genomes were visualized using Bandage^44^. Finally, the obtained assemblies were annotated using Prokka^45^ (v1.14.6). The same was done for genomes obtained from public databases. Based on these annotations, a core genome was defined using Roary^46^ (v3.13.0), and a phylogenic tree was generated and visualized using IQ-TREE^47^ (v2.3.6) and iTOL^48^ (v6.9.1) respectively. Clonal complex designations were determined using PubMLST^49^. Trimmomatic, Porechop, Unicycler, Prokka, Roary and IQ-TREE were used on the Galaxy Europe server.

### Bacterial isolate for in vitro and in vivo studies

The bacterial strain used in this in vitro and in the in vivo study was a clinical *S. aureus* strain: JAR060131, available at the Swiss culture Collection (Wadenswil, Switzerland; accession number CCOS 890). The strain was cultured from a patient with a musculoskeletal infection and is sensitive to vancomycin.

### SAC preparation

The preparation of SAC was performed as follows^13^: an overnight culture of *S. aureus* JAR was resuspended PBS (Invitrogen) to obtain a suspension containing approximately 10 CFU in 5 μl. The suspension was embedded between two layers of rat collagen I solution (Corning). After collagen polymerization in a 37 °C incubator with 5% CO_2_, 100 μl of pooled human plasma (Regional Blood Donation Service SRK Graubünden) was added and the samples were incubated for a total of 15 h. After removal of the plasma, mature SACs were challenged by applying 200 µl of the selected treatment: PBS containing 10 µM SAK2, 10 µM RODI∆ami, 10 µM FL-028 or 10 μg ml^−1^ vancomycin (Roth AG). Treatments were reapplied every 10 h. Following treatment, SACs were mechanically disrupted, serially diluted and plated for CFU quantification. CLSM was performed using fibrin and bacterial viability staining as described below^14^. All experiments were performed in triplicate (*n* = 3), with each having at least three biological replicates. Error bars indicate a standard deviation from the mean.

### Biofilm preparation

A bacterial suspension with an OD_600_ of 0.1 was prepared from an overnight culture of *S. aureus* JAR. Titanium disks (6 × 2 mm Ti6Al4V Grade 23 ELI) were placed at the bottom of a 48-well plate and 100 µl of the bacterial suspension was added onto each disk. Subsequently, 900 µl of TSB supplemented with 1% human plasma was added. The discs were incubated at 37 °C in static conditions for 48 h with a media change after 24 h. After removal of the medium, mature biofilm were challenged by applying 200 µl of the selected treatment: PBS containing 10 µM SAK2, 10 µM RODI∆ami, 10 µM FL-028 or 10 μg ml^−1^ vancomycin (Roth AG). Treatments were repeated every 10 h. Following treatment, biofilms were removed from titanium discs by sonication and vortexing, serially diluted and plated for CFU enumeration. Additional samples were processed for CLSM^50^. All experiments were performed in triplicate (*n* = 3), with each having at least three biological replicates. Error bars indicate a standard deviation from the mean.

### Sample imaging

The Live/Dead BacLight bacterial viability kit (Invitrogen) was used to stain SAC and biofilms, following the manufacturer’s instructions. In brief, 500 μl of staining solution containing Syto9 (to label live cells) and propidium iodide (to label dead cells) was applied to each sample. For fibrin visualization, exogenous fibrinogen labelled with Alexa Fluor 647 (Molecular Probes) in 2 mM CaCl_2_ was added to human plasma. CLSM was performed using a Zeiss LSM 800 microscope or a Leica TCS SP8 X microscope. Fluorescent images were captured with a Zeiss Axio Vert.A1 microscope (Zeiss), and image processing was conducted using ZEN (blue edition) software (Zeiss). Samples for scanning electron microscopy were fixed in McDowell’s fixative, dehydrated through a graded ethanol series, paraffin-embedded, sectioned, deparaffinized, air-dried, sputter-coated with gold/palladium and imaged using a Hitachi S-4700 field emission scanning electron microscope.

### In vitro viability assay

Telomerase-immortalized human foreskin fibroblasts (hTERT-BJ1) were purchased from Clontech and cultured at 37 °C in a humidified atmosphere containing 5% CO_2_ in Dulbecco’s modified Eagle medium (Gibco, Thermo Fisher) supplemented with 1 g l^−1^ glucose (low glucose), 10% fetal bovine serum (Corning), 100 U ml^−1^ penicillin and 100 μg ml^−1^ streptomycin (Gibco, Thermo Fisher). Cell line authentication was not independently performed by the authors.

For viability assessment, a CellTiter-Blue assay (Promega) was performed over a total experimental period of 170 h. Cells were seeded in 96-well plates at a density of 1 × 10^4^ cells per well in complete medium (Dulbecco’s modified Eagle medium + 10% fetal bovine serum + 1% penicillin/streptomycin). After overnight attachment, cells were treated with 10 µM SAK2, 10 µM RODI∆ami or 10 µM FL-028. Control cells received complete medium only. Cell viability was assessed at 24, 72 and 170 h post-treatment. At 72 h, culture medium was refreshed with fresh compound-containing medium. At each timepoint, medium was replaced with fresh complete medium containing CellTiter-Blue reagent, and cells were incubated for 2 h at 37 °C/5% CO_2_ according to the manufacturer’s instructions. Absorbance was measured at 570 nm with a reference wavelength of 600 nm using a plate reader (TECAN). Each condition was tested in six technical replicates. Background signal from wells containing medium and reagent only was subtracted from all measurements. Data were normalized to the untreated control condition at each corresponding timepoint and expressed as relative cell viability.

### In vivo animal model

Sixty-four female C57BL/6N mice, aged 19–28 weeks, were included in the in vivo study (non-infected healing study, and infected efficacy study), using a previously characterized mouse model^51^. The animal study was approved by the relevant cantonal authorities (Amt für Lebensmittelsicherheit und Tiergesundheit Graubünden) with the approval numbers 01/2023, 17E/2023 and 36E/2023 and was carried out in a research facility accredited by the Association for Assessment and Accreditation for Laboratory Animal Care International. Sample sizes were selected based on previous experience with the models used, published studies using the same experimental systems, and expected biological variability. No formal a priori power calculation was performed. To assess the influence of FL-028 on bone healing in non-infected mice, 20 mice underwent surgery to create a femoral osteotomy fixed with a four-hole MouseFix plate (RISystem). Treatment with FL-028, vancomycin and their combination or saline was administered locally from day 5 to day 18 (*n* = 10 per group). At day 21 post operation, the animals were euthanized and the femur were processed for mechanical testing (*n* = 12) or histology (*n* = 8) (schematically represented in Extended Data Fig. 8a). For the model of fracture-related infection, 44 mice underwent the same initial surgery as described above. At the time of surgery, the osteotomy was inoculated with 10^4^ CFU of *S. aureus* JAR. The infection was allowed to develop for five days, after which revision surgery was performed. During this procedure, the wound was debrided and flushed, and samples were collected to assess infection progression. Both the wash solution and debrided tissue surrounding the infected implant were analysed for bacterial quantification. Treatment was administered from day 5 to day 18 (Extended Data Fig. 8b). FL-028 was administered once daily as a 50-µl local injection at the osteotomy site (10 µM) from day 5 to day 18, with *n* = 8 mice per group for bacteriological analyses and *n* = 2–4 for histology. The animals received one daily injection FL-028 or both unfused enzymes locally at the osteotomy or subcutaneous injections of 2.75 mg vancomycin^51^, or the combination of FibriLysin and vancomycin at the same doses. A 3-day washout period was included to allow enzymes and antibiotics to clear, before the mice were euthanized and analysed on day 21 post-initial surgery.

### Animal housing and welfare

Animals were group-housed in individually ventilated cages (XJ, Allentown) with a 12-h light/dark regimen, between 20 °C and 24 °C and 30–70% relative humidity and fed with extrudate (3436, Kliba Nafag). Mice were acclimatized to the housing conditions for at least 4 weeks and randomly allocated to their respective groups. For both surgeries, mice were anaesthetized using Sevoflurane (Baxter) in oxygen. Analgesia was provided by Tramadol in the drinking water (200 mg l^−1^) from 1 day pre-operation until 5 days post-revision, as well as Carprofen injection (5 mg kg^−1^) after anaesthesia induction. For subsequent daily local treatments, mice were put under short general anaesthesia using Sevoflurane. Fifty microlitres of FibriLysin solution was injected locally at the osteotomy with an insulin syringe. One group received 2.75 mg vancomycin (Sandoz) twice daily subcutaneously^51^. In total, 80 mice were enrolled in the study. Sixteen of them (20%) needed to be excluded for reaching the humane endpoint. Most of the exclusions were due to wound dehiscence or fracture of the operated bone/implant failure later in the study. Histological assessment and surgical evaluation were performed by investigators blinded to treatment allocation.

### Histology sections and µCT analysis

Animals were euthanized after 21 days, the implants were first removed, and the femurs were fixed in formalin and decalcified with ethylenediaminetetraacetic acid decalcification solution and embedded in paraffin. Paraffin-embedded samples (5 μm in thickness) were stained with haematoxylin and eosin (H&E) and Picro-Mallory trichrome (PM) stain to stain bacteria and fibrin by following a previously described protocol^52^. Stained paraffin sections were dehydrated with three absolute ethanol steps, cleared two times in pure xylene for 5 min, and mounted with Eukitt mounting medium (Sigma-Aldrich). The operated leg was scanned using computed tomography immediately after inoculation surgery, after revision surgery and post-mortem. Bone volume within and around the defect was monitored using the cone-beam in vivo microCT scanner VivaCT80 (SCANCO Medical AG). Animals were scanned under sevoflurane anaesthesia immediately following surgery, at 5 days post-operatively and after euthanasia on day 21. To prevent movement during the scan, the femur was mounted in a custom-made acrylonitrile–butadiene–styrene holder. A 3.74-mm region, centred on the femoral defect, was scanned with a 21.5-mm field of view. The X-ray source was set to 70 kV and 114 μA, with a 0.5-mm aluminium filter to reduce beam-hardening artefacts. In total, 1,000 projections were captured over a 180° rotation with a 300-ms integration time per projection. The raw projection data were then reconstructed across an image matrix of 2,066 × 2,066 pixels with an isotropic voxel size of 10.4 μm, expressed in terms of voxel values corresponding to bone mineral density in mg hydroxyapatite cm^−^^3^. All image analyses were performed using Amira 2021.1 (FEI SAS, part of Thermo Fisher Scientific). The postoperative scans served as baseline scans to define two regions of interest (ROIs). The osteotomy gap was segmented via interpolation between both osteotomy lines to determine the initial gap size, while the bone outside of the defect site included an additional 0.42 mm on either side of the osteotomy region. Subsequent scans were registered to the baseline scans using rigid registration. The resulting transformation matrix was used to apply the ROI to the respective scans, ensuring the evaluation of the same region over time. Before evaluation, the scans were Gaussian-filtered (standard deviation = 2, kernel size = 3) to reduce noise. Bone was segmented automatically with a threshold of 650 mg hydroxyapatite cm^−^^3^, and bone volume within the ROIs was computed using direct voxel counting methods. All image processing and analysis were performed using custom TCL scripts in Amira.

### Reporting summary

Further information on research design is available in the Nature Portfolio Reporting Summary linked to this article.

## Supplementary information

Reporting Summary

Supplementary Tables 1 and 2 Supplementary Tables 1 and 2.

## Source data

Source Data Fig. 1 Full-size microscopy images for Fig. 1c.

Source Data Fig. 2 Full-size microscopy images for Fig. 2a,b.

Source Data Fig. 2 Raw data for Fig. 2c,d.

Source Data Fig. 3 Source data for Fig. 3.

Source Data Fig. 3 Raw data for Fig. 3.

Source Data Fig. 4 Full-size μCT radiographs, μCT reconstructions and histology images for Fig. 4d–f.

Source Data Fig. 4 Raw data for Fig. 4.

Source Data Fig. 5 Full-size histology and FISH images for Fig. 5c.

Source Data Fig. 5 Raw data for Fig. 5.

**Extended Data Fig. 1:**
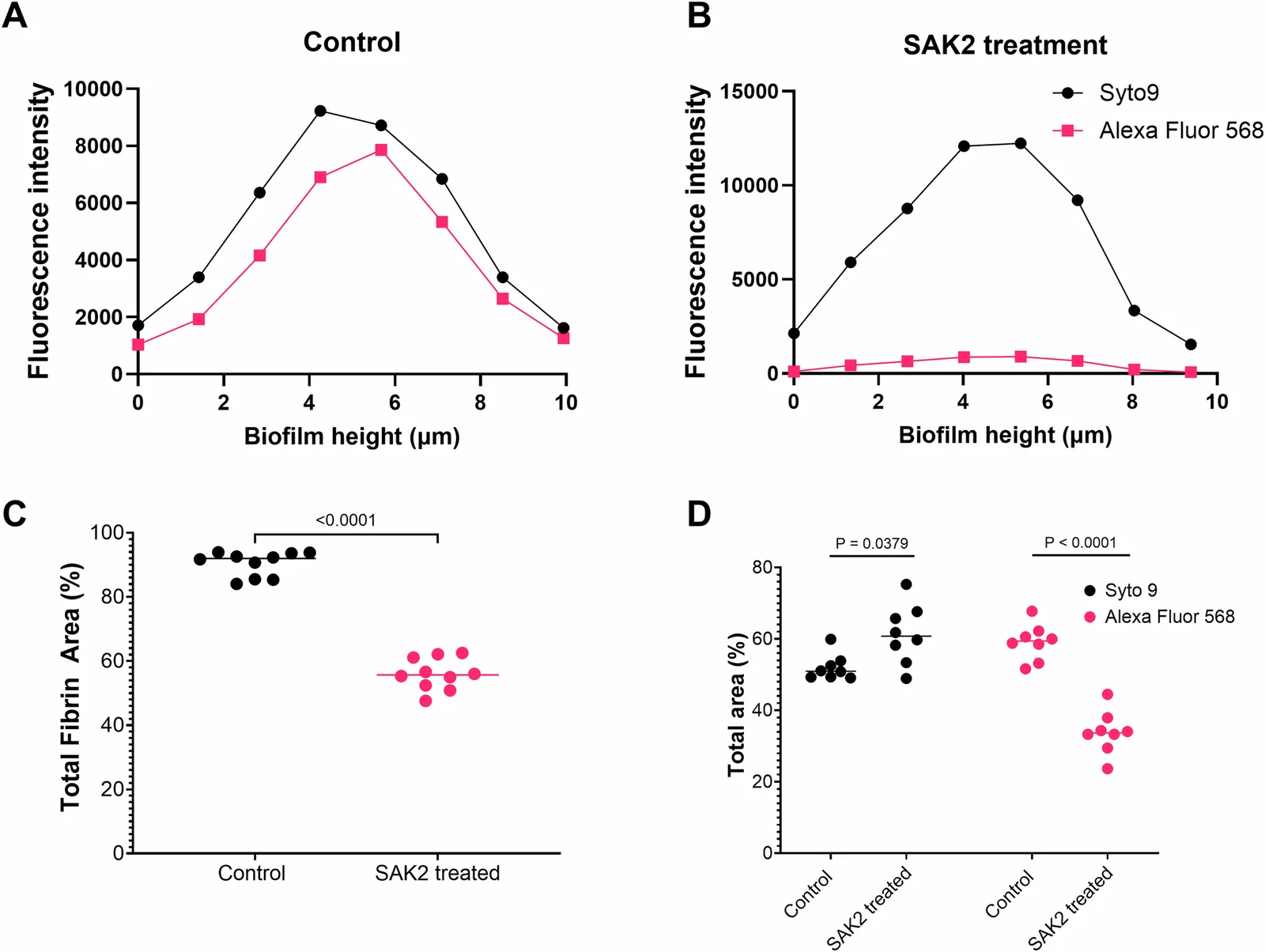
Fibrinolytic activity of SAK2 *in vitro*. Mature biofilms grown on titanium discs in TSB medium were exposed to PBS (**A**) or 10 µM SAK2 (**B**) for 24 h. Data from representative CLSM images from independent biofilm samples are shown. The signal intensity from Syto9 (live bacteria) and Alexa Fluor 568 (fibrin) was measured throughout the biofilm thickness. (**C**) Total fibrin area was quantified from CLSM images. Each data point represents one independently imaged biofilm sample from a set of 8 biological replicate experiments. Statistical analysis was performed using a two-sided unpaired t-test (P < 0.0001). (**D**) Surface coverage by bacterial cells (Syto9) and fibrin area (Alexa Fluor 568) were quantified from CLSM images. Each data point represents one independently imaged biofilm sample from a set of 8 biological replicate experiments. Statistical analysis of Syto9 signal was performed using a two-sided Mann-Whitney test (U = 12, P = 0.0379). Statistical analysis of Alexa Fluor 568 signal was performed using a two-sided unpaired t-test (t = 9.102, d.f. = 14, P < 0.0001). Source data

Source Data Extended Data Fig. 1 Raw data for Extended Data Fig. 1.

**Extended Data Fig. 2:**
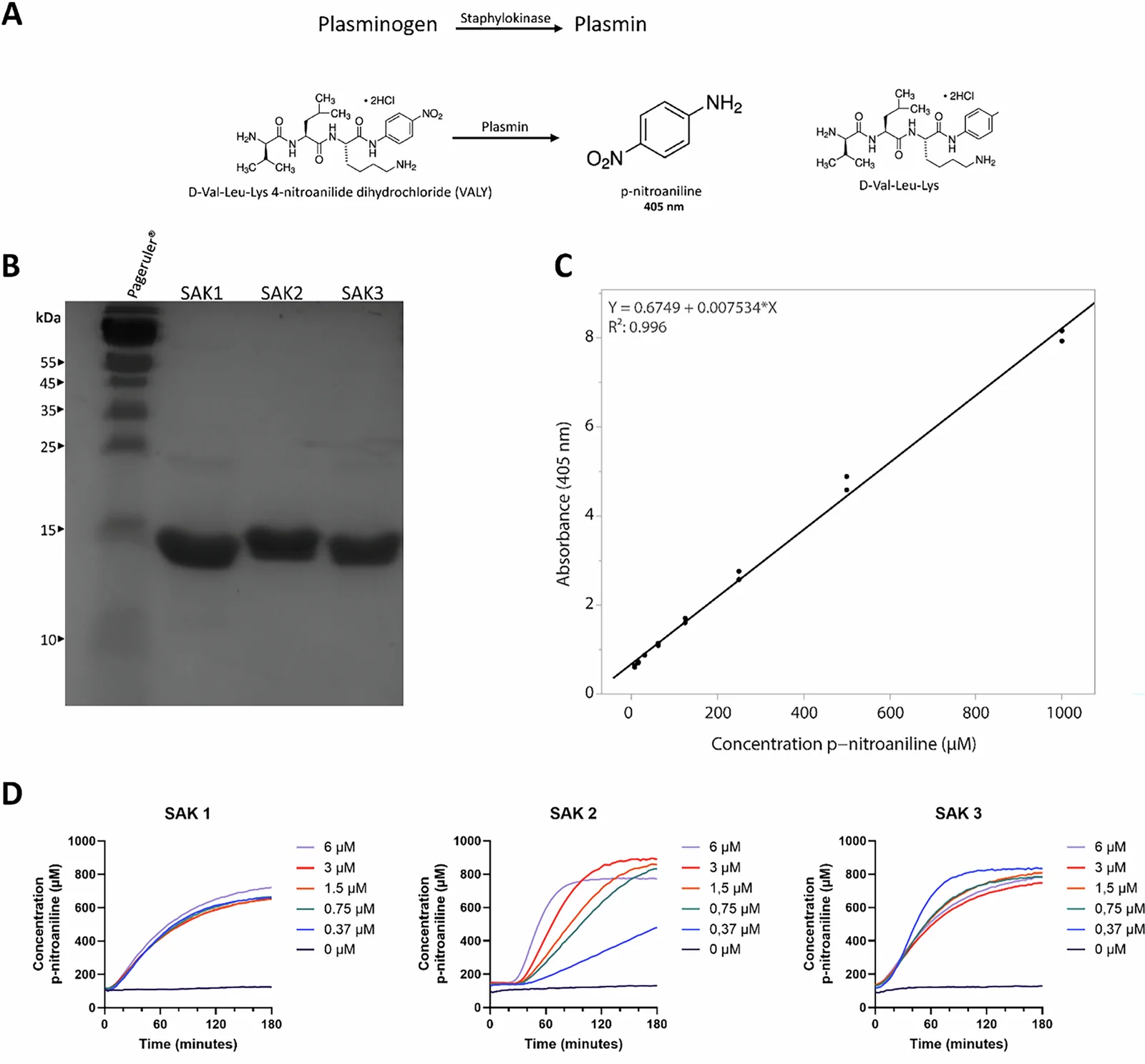
Validation of *in vitro* plasminogen activating activity of recombinantly expressed SAKs. (**A**) Plasminogen is converted into plasmin, which then cleaves the peptide substrate VALY, resulting in the release of p-nitroaniline, detectable by absorbance at 405 nm. (**B**) Representative 12% SDS-PAGE gel showing recombinantly expressed SAKs following Ni-NTA purification, illustrating the purity of the recombinant proteins used in this study. Similar results were obtained in three independent protein expression and purification experiments. (**C**) A linear relationship was established between the concentration of p-nitroaniline and absorbance at 405 nm, allowing quantification of the plasmin-mediated conversion of VALY to p-nitroaniline. (**D**) SAK-mediated plasminogen activation assay with the three recombinant SAKs. All three SAKs activated human plasminogen to plasmin. Data are presented as mean ± SD from n = 3 independent biological replicates, each analysed in triplicate technical replicates. For SAK2, a dose-dependent response was observed, whereas this was not observed for SAK1 or SAK3. Source data

Source Data Extended Data Fig. 2 Unprocessed blots for Extended Data Fig. 2.

**Extended Data Fig. 3:**
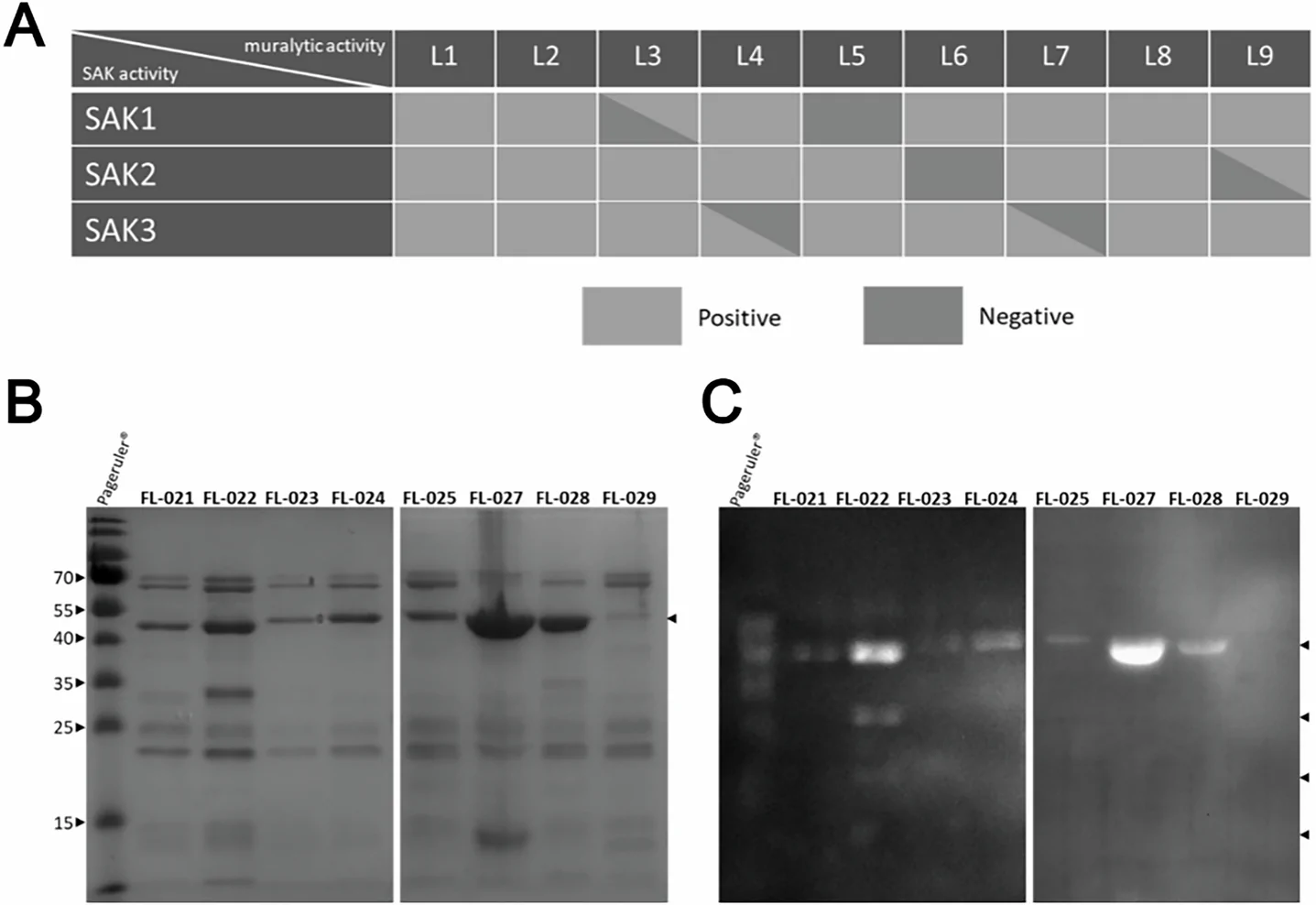
Development and screening of the FibriLysin library. **(A)** Summary of muralytic and plasminogen-activating activity screening results obtained for the 27 FibriLysin constructs generated by combining three staphylokinases (SAK1-3) with nine linker variants and the antistaphylococcal endolysin RODIΔami. Twenty-one of the 27 constructs retained both activities, corresponding to a positive hit rate of 78%. **(B)** Representative SDS-PAGE analysis of selected hit variants following benchtop Ni-NTA purification from 500 mL expression cultures. Protein bands corresponding to full-length FibriLysins were detected at approximately 50 kDa. **(C)** Representative anti-His Western blot analysis of the same purified proteins confirming expression and structural integrity of the selected FibriLysin variants. While FL-022 and FL-028 displayed strong antibacterial activity, FL-022 showed evidence of protein fragmentation. Source data

**Extended Data Fig. 4:**
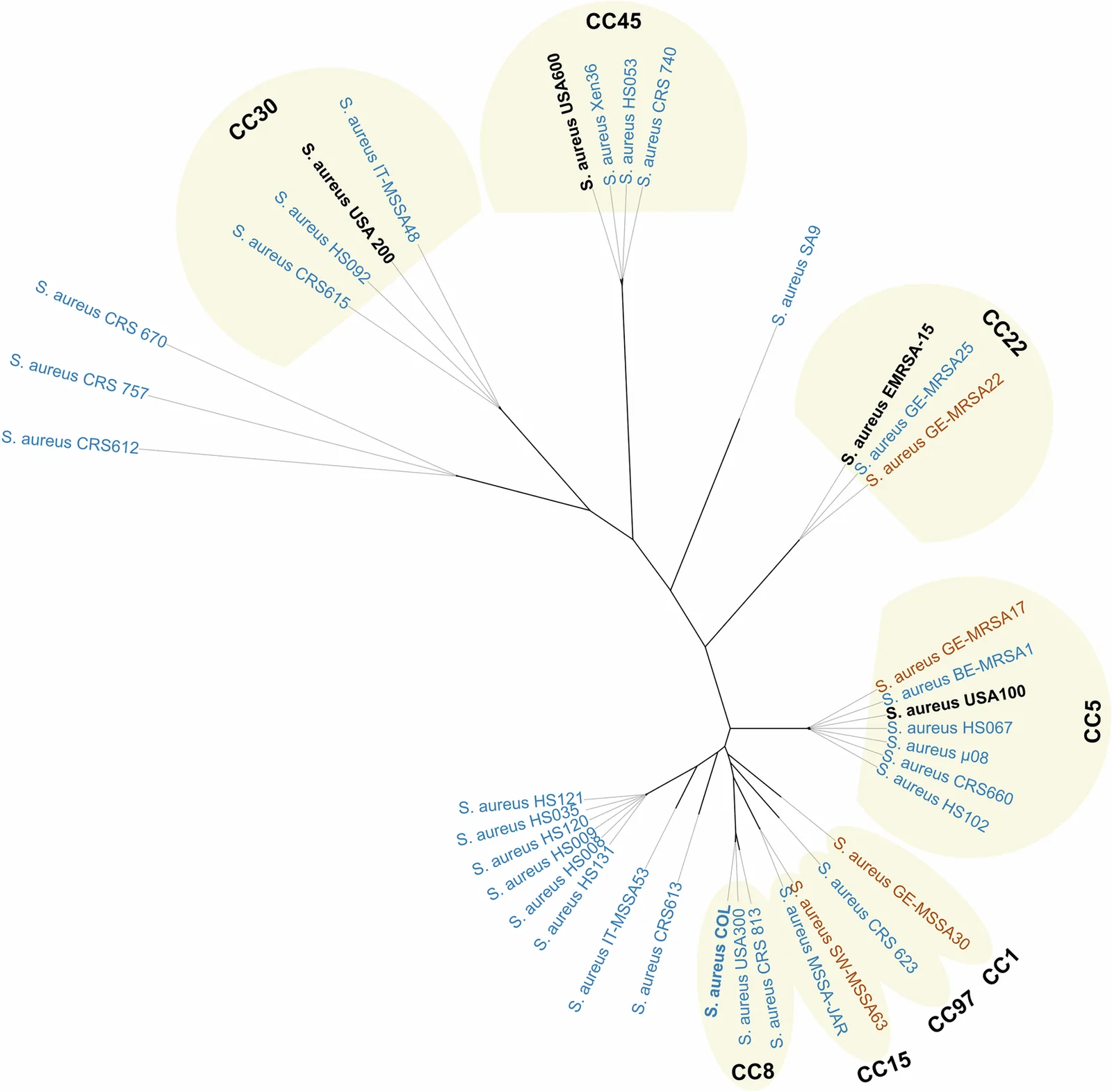
FL-028 is active against a diverse set of clinical *S. aureus* isolates. Antibacterial activity was tested with a minimal inhibitory concentration (MIC) assay. Bacterial isolates included both clinical and laboratory strains (n = 33) including drug-resistant isolates, sourced as indicated in Table S4. Growth of bacterial isolates indicated in green could be inhibited whereas strains in red were insensitive to FL-028. Strains indicated in black were used as reference strains for their respective clonal complex when defining the core genome. Clonal complexes of common healthcare-acquired and community acquired MRSA lineages^55^ were represented (CC1, CC5, CC8, CC22, CC45) in the tested set.

Source Data Extended Data Fig. 3 Unprocessed blots for Extended Data Fig. 3.

**Extended Data Fig. 5:**
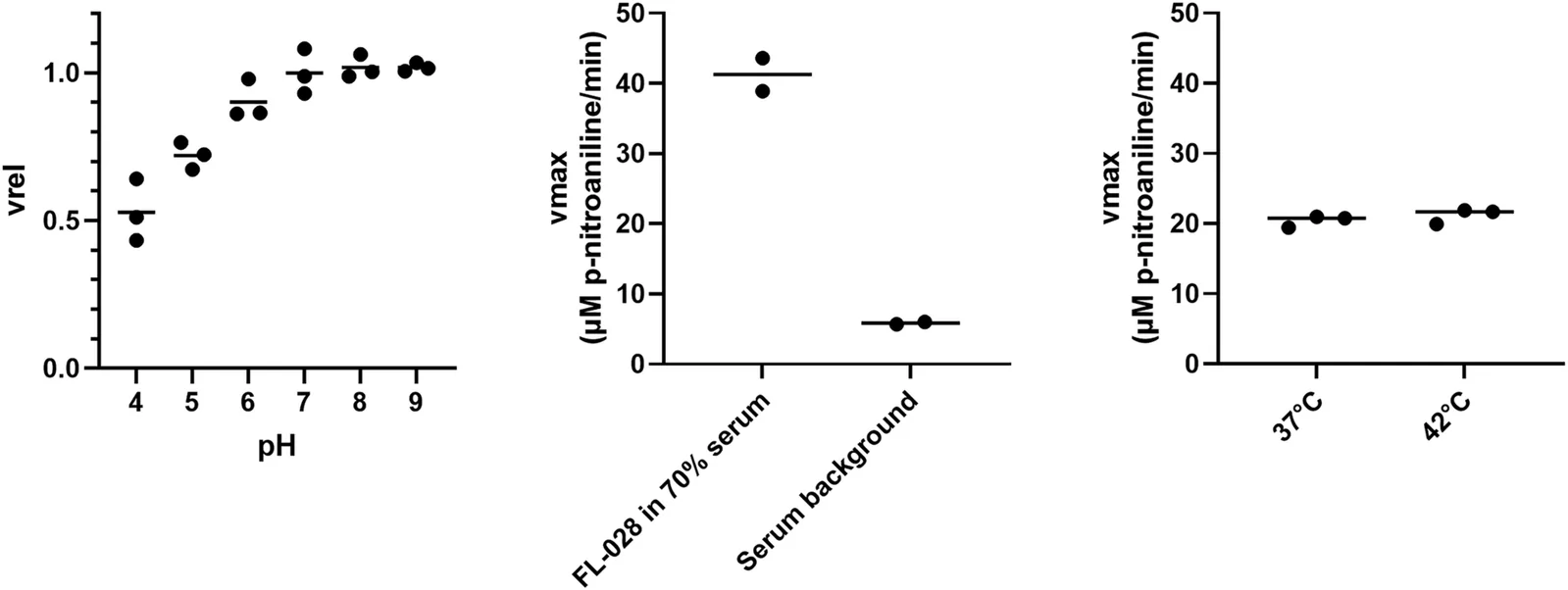
Plasminogen-activating activity of FL-028 in a range of different pHs (left), in heat-inactivated human serum (middle) and at elevated temperature (right). Data are presented as mean ± SD from n = 3 independent biological replicates, each analysed in triplicate technical replicates. At lower pH, a reduction in either plasmin activity or plasminogen activation is observed up to 47% of the activity at pH = 7.4. At higher pHs, no reduction in enzyme or enzyme-activating activity is observed. As this *in vitro* plasminogen-activating assay is an indirect assay, in which the conversion of a chromogenic substrate by plasmin is measured, reduced activity at lower pH could also be attributed to reduced enzymatic activity of plasmin itself, having a pH optimum of 7.5^56^. Nonetheless, 50% of the conversion velocity at optimal pH was still achieved at pH = 4.0. (Middle) Activity in 70% heat-inactivated serum was observed in vitro, indicating retention of plasminogen-activating activity in serum-containing conditions. (Right) When incubated at 42°C, mimicking a feverish temperature, FL-028 retains its activity when compared to the same conditions at 37°C. Source data

Source Data Extended Data Fig. 5 Raw data for Extended Data Fig. 5.

**Extended Data Fig. 6:**
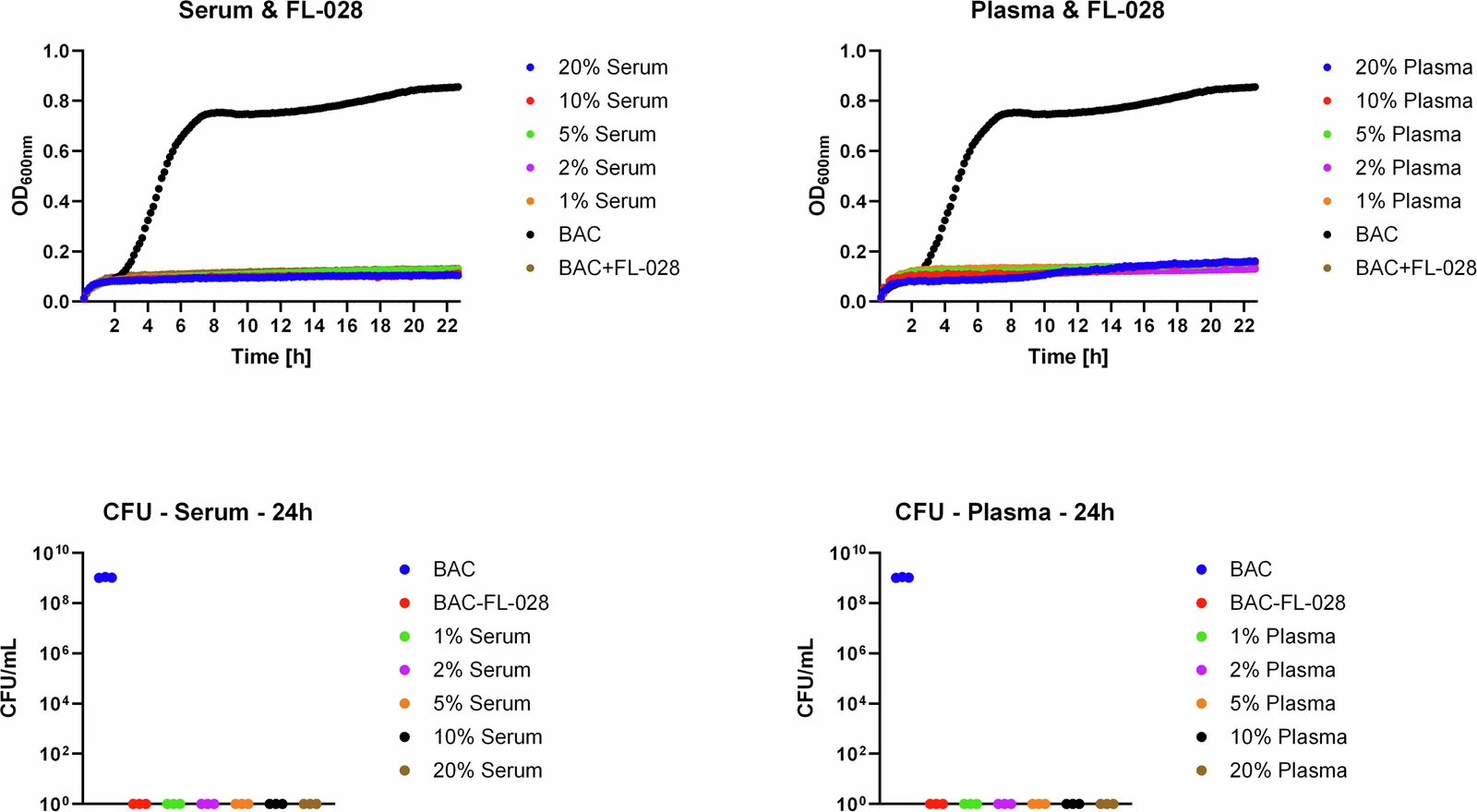
FL-028 retains antibacterial activity against *S. aureus* in the presence of serum and plasma. *S. aureus* cultures (OD600 = 0.001) were treated with FL-028 at 10× MIC in the presence of increasing concentrations (1–20%) of serum or plasma. Bacterial growth was monitored by measuring the OD600 over 24 h. Untreated bacteria (BAC) and FL-028-treated bacteria without serum or plasma (BAC + FL-028) were used as controls. After 24 h, viable bacteria were quantified as CFU/mL. Technical replicates were analysed within a single experiment. Each data point represents one technical replicate. FL-028 inhibited bacterial growth across all tested serum and plasma concentrations. Source data

Source Data Extended Data Fig. 6 Raw data for Extended data Fig. 6.

**Extended Data Fig. 7:**
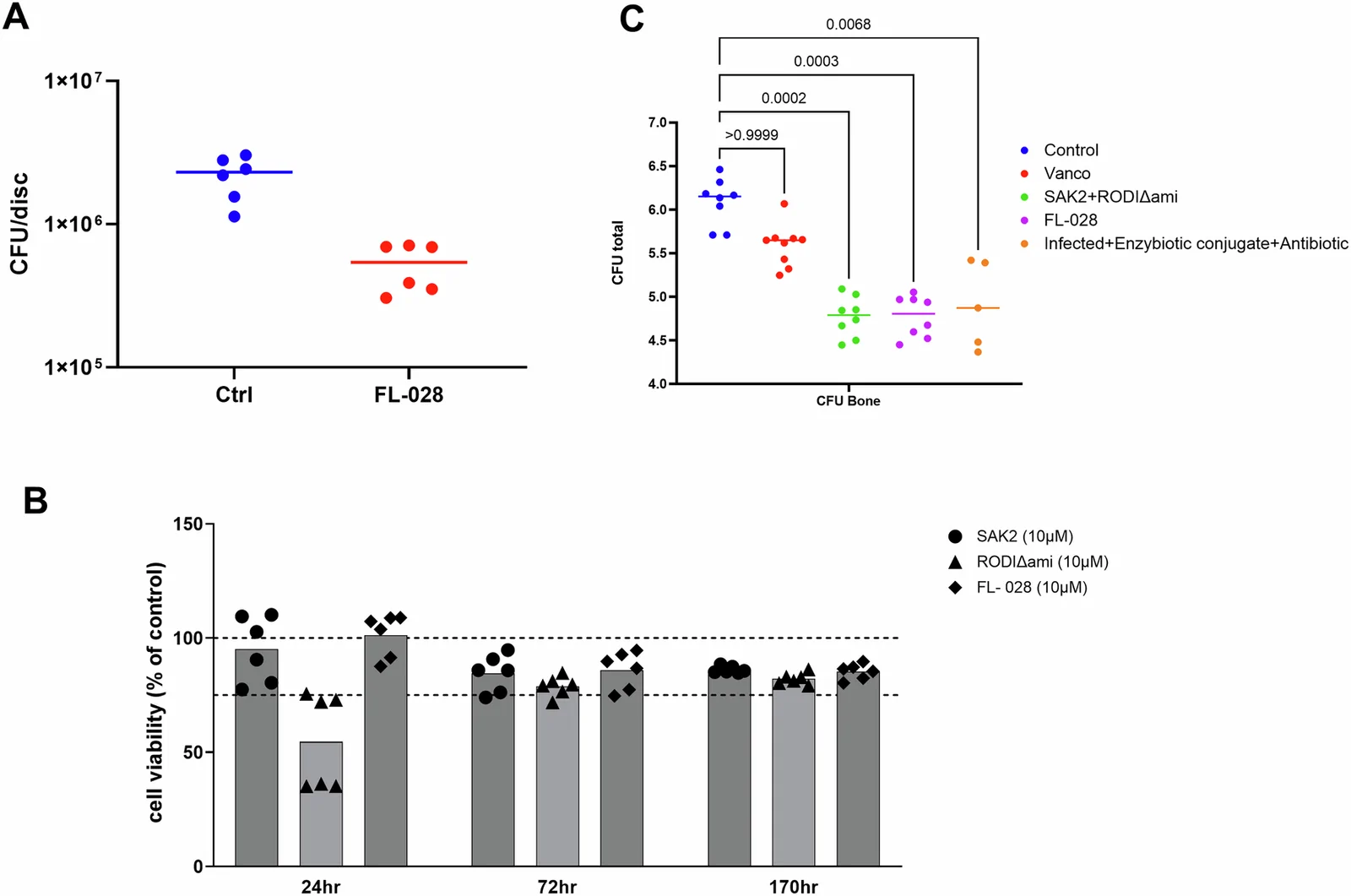
Additional efficacy and safety characterization of FL-028. (**A**) A 48 h S. epidermidis biofilm was treated for 24 h with 10 µM FL-028 or left untreated (Ctrl). Viable bacteria were quantified as CFU per disc. Six technical replicates were analysed per treatment group within a single experiment. Each data point represents one technical replicate. (**B**) hTERT-BJ1 human fibroblasts were treated with 10 µM SAK2, RODIΔami or FL-028 for 24, 72 and 170 h. Cell viability was assessed using the CellTiter-Blue assay and expressed relative to untreated control cells. Six technical replicates were analysed for each condition within a single experiment. Each data point represents one technical replicate. Bars indicate mean values. (**C**) Bacterial burden in an in vivo fracture-related infection model following treatment with FL-028, unfused enzymes, vancomycin or combination therapy. Mice with established fracture-related Staphylococcus aureus infection were treated for 14 days with daily administrations of either FL-028 (10 µM, 50 µL, local injection at the osteotomy site), an equimolar mixture of the unfused component enzymes administered locally, vancomycin alone (2.75 mg, subcutaneous injection), or vancomycin in combination with local FL-028 administration. At the end of the treatment period, animals were sacrificed and bacterial burden within the infected bone was quantified as colony-forming units (CFU). Each data point represents one animal (biological replicate; n = 8 mice per group, except n = 5 mice for the FL-028 plus vancomycin combination treatment group). Statistical analysis was performed using a two-sided Kruskal-Wallis test followed by Dunn’s multiple-comparisons test. Adjusted P values are shown on the graph. Source data

**Extended Data Fig. 8:**
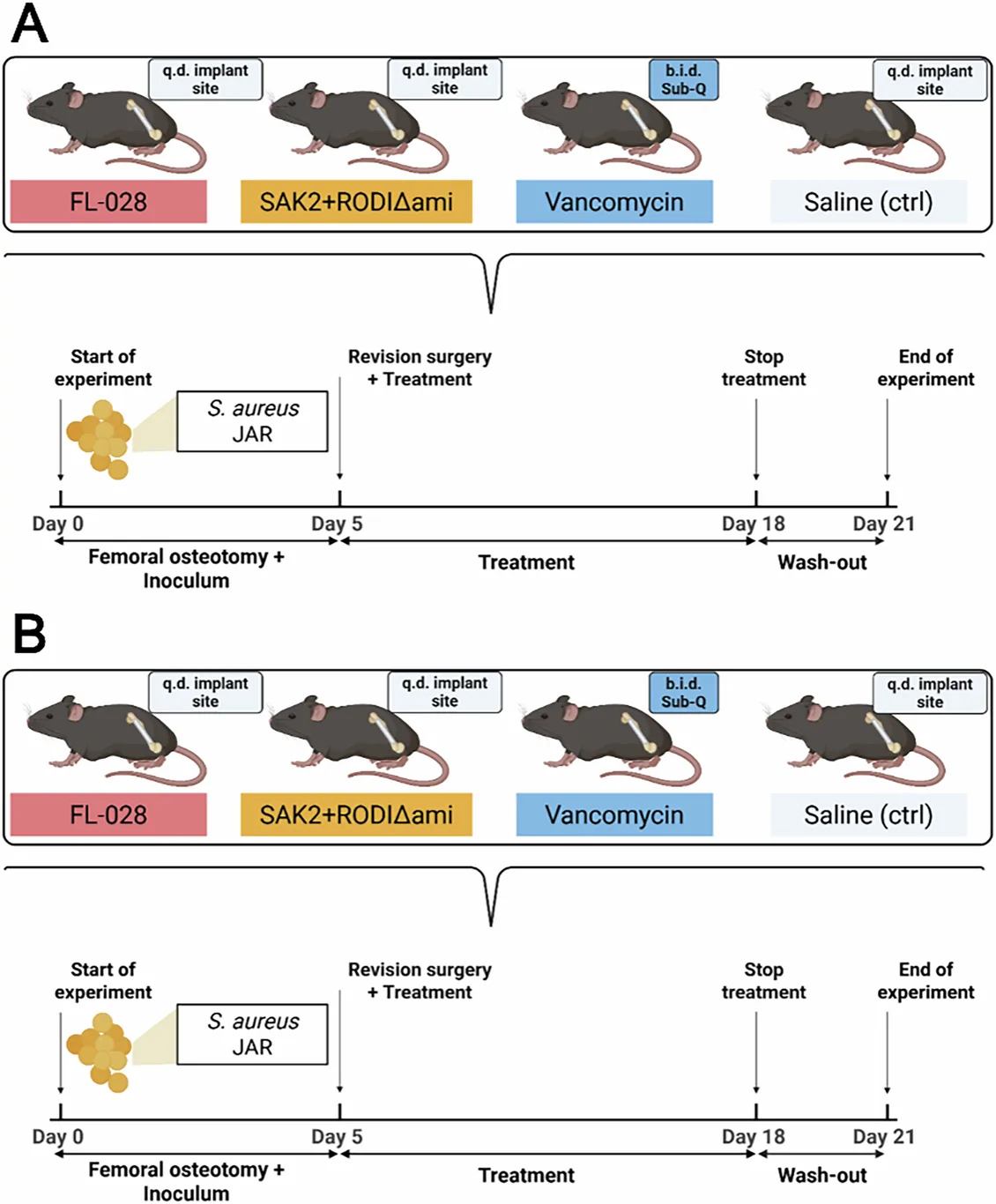
Schematic representation of the murine studies used to evaluate the safety profile (A) and therapeutic efficacy (B) of FL-028. (**A**) Bone healing study. Mice underwent femoral osteotomy and were assigned to receive either FL-028 or saline control (n = 12 per group). Treatment commenced on day 5 after surgery and continued until day 18. Animals received daily local injections of FL-028 (10 µM) or saline at the implant site in a final volume of 50 µL. Following a 3-day washout period, animals were euthanized on day 21 for analysis. (**B**) Fracture-related infection study. Mice underwent femoral osteotomy with *S. aureus* inoculation on day 0, followed by revision surgery and treatment initiation on day 5. Animals received daily local injections of FL-028 (10 µM), an equimolar mixture of SAK2 and RODIΔami, or saline control (50 µL at the implant site), or vancomycin administered subcutaneously twice daily. Treatment continued until day 18, followed by a 3-day washout period prior to endpoint analysis on day 21.

Source Data Extended Data Fig. 7 Raw data for Extended Data Fig. 7.

## Data Availability

The data underlying this study are available in the Article and its Supplementary Information. Genome sequencing data generated in this study are available from NCBI under BioProject accession number PRJNA1481042. Newly generated plasmids and recombinant constructs are available from the corresponding authors upon reasonable request. Requests for materials will typically be addressed within 30 days. Distribution of materials may require completion of an appropriate Material Transfer Agreement. Source data are provided with this paper.

## Extended data

**Extended Data Table 1:** Library of SAK, RODIΔAMI and Linkers assembled by Versatile platform

Name	Protein sequence
**SAK1**	MSSSFDKGKYKKGDDASYFEPTGPYLMVNVTGVDSKGNELLSPHYVEFPIKPGTTLTKEKIEYYVEWALDATAYKEFRVVELDPSAKIEVTYYDKNKKKEETKSFPITEKGFVVPDLSEHIKNPGFNLITKVVIEK
**SAK2**	MATYDHKQIKIGESRDYQEIVGPHLIVNVTGVDKNGNDKLHPKFMEFHIKPGQVLNKKEITKYVEWTLDGTAYNKYRVVDFAQGSKVEVTYFSKTEKRNVTQSFPITDKGFVVPDLSEHTTNPGYTLVTNVIIEEKKSKK
**SAK3**	MSSSFDKGKYKKGDDASYFEPTGPYLMVNVTGVDGKRNELLSPRYVEFPIKPGTTLTKEKIEYYVEWALDATAYKEFRVVELDPSAKIEVTYYDKNKKKEETKSFPITEKGFVVPDLSEHIKNPGFNLITKVVIEKK
**RODIΔAMI**	MAKTQAEINKRLDAYAKGTVDSPYRIKKATSYDPSFGVMEAGAIDADGYYHAQCQDLITDYVLWLTDNKVRTWGNAKDQIKQSYGTGFKIHENKPSTVPKKGWIAVFTSGSYQQWGHIGIVYDGGNTSTFTILEQNWNGYANKKPTKRVDNYYGLTHFIEIPVKAGATSSSTIVKDGKTSSASTPATRPVTGSWKKNQFGTWYKPESATFVNGNQPIITRIGSPFLNAPIGGNLPAGATIVYDEVCIQAGHIWIGYNAYNGNRVYCPVRTCQGVPPNHIPGVAWGVFKG
**L 1**	AGAGAG
**L 2**	GAGAGAGAGAGAGA
**L 3**	LSRFFHAEL
**L 4**	VFNQRKEHKGYMLA
**L 5**	IPQGRSHPVQPYPGAF
**L 6**	PAVPPP
**L 7**	EAAAKEAAKEAAK
**L 8**	AGYGKAGGTVTPTPNT
**L 9**	EAAAKEAAAKEAAAK
*Fibrilysin library combinations (markerless fusion between indicated domains)*	
**FL-011**	SAK1-LINKER1-RODIΔAMI
**FL-012**	SAK1-LINKER2-RODIΔAMI
**FL-013**	SAK1-LINKER3-RODIΔAMI
**FL-014**	SAK1-LINKER4-RODIΔAMI
**FL-015**	SAK1-LINKER5-RODIΔAMI
**FL-016**	SAK1-LINKER6-RODIΔAMI
**FL-017**	SAK1-LINKER7-RODIΔAMI
**FL-018**	SAK1-LINKER8-RODIΔAMI
**FL-019**	SAK1-LINKER9-RODIΔAMI
**FL-021**	SAK2-LINKER1-RODIΔAMI
**FL-022**	SAK2-LINKER2-RODIΔAMI
**FL-023**	SAK2-LINKER3-RODIΔAMI
**FL-024**	SAK2-LINKER4-RODIΔAMI
**FL-025**	SAK2-LINKER5-RODIΔAMI
**FL-026**	SAK2-LINKER6-RODIΔAMI
**FL-027**	SAK2-LINKER7-RODIΔAMI
**FL-028**	SAK2-LINKER8-RODIΔAMI
**FL-029**	SAK2-LINKER9-RODIΔAMI
**FL-031**	SAK3-LINKER1-RODIΔAMI
**FL-032**	SAK3-LINKER2-RODIΔAMI
**FL-033**	SAK3-LINKER3-RODIΔAMI
**FL-034**	SAK3-LINKER4-RODIΔAMI
**FL-035**	SAK3-LINKER5-RODIΔAMI
**FL-036**	SAK3-LINKER6-RODIΔAMI
**FL-037**	SAK3-LINKER7-RODIΔAMI
**FL-038**	SAK3-LINKER8-RODIΔAMI
**FL-039**	SAK3-LINKER9-RODIΔAMI

**Extended Data Table 2:** MIC values, sensitivities and source of *S. aureus* isolates challenged with FL-028

Strain name	CC type	MIC	Sensitivity	Source	Reference
GE-MSSA30	CC1	Insensitive	Not sensitive	Orthopaedic device related infection	Ponce Benavente et al., 2024
GE-MRSA17	CC5	Insensitive	Not sensitive	Orthopaedic device related infection	Ponce Benavente et al., 2024
BE-MRSA1	CC5	0.25 µM	Sensitive	Fracture related infection	Ponce Benavente et al., 2024
HS067	CC5	1.25 µM	Sensitive	Hidradenitis Suppurativa	Bens et al., 2023
Mu08	CC5	0.25 µM	Sensitive	Orthopaedic device related infection	Hofstee et al., 2020
CRS660	CC5	0.31 µM	Sensitive	Chronic rhinosinusitis	Onsea et al., 2021
HS102	CC5	1.25 µM	Sensitive	Hidradenitis Suppurativa	Bens et al., 2023
COL	CC8	0.25 µM	Sensitive	Laboratory strain	Ponce Benavente et al., 2024
USA300	CC8	0.5 µM	Sensitive	Clinical isolate	King et al., 2006
CRS813	CC8	Insensitive	Not sensitive	Chronic rhinosinusitis	Onsea et al., 2021
SW-MSSA63	CC15	Insensitive	Not sensitive	Fracture-related Infection	Ponce Benavente et al., 2024
MSSA-JAR	CC15	0.25 µM	Sensitive	Orthopaedic device related infection	Ponce Benavente et al., 2024
GE-MRSA25	CC22	0.25 µM	Sensitive	Fracture-related Infection	Ponce Benavente et al., 2024
GE-MRSA22	CC22	Insensitive	Not sensitive	Fracture-related Infection	Ponce Benavente et al., 2024
CRS615	CC30	0.62 µM	Sensitive	Chronic rhinosinusitis	Onsea et al., 2021
HS092	CC30	0.62 µM	Sensitive	Hidradenitis Suppurativa	Bens et al., 2023
IT-MSSA48	CC30	0.25 µM	Sensitive	Pacemaker-related Infection	Ponce Benavente et al., 2024
Xen36	CC45	0.12 µM	Sensitive	Engineered clinical isolate	
HS053	CC45	0.31 µM	Sensitive	Hidradenitis Suppurativa	Bens et al., 2023
CRS740	CC45	0.15 µM	Sensitive	Chronic rhinosinusitis	Onsea et al., 2021
CRS623	CC97	0.62 µM	Sensitive	Chronic rhinosinusitis	Onsea et al., 2021
CRS670	N/A	0.62 µM	Sensitive	Chronic rhinosinusitis	Onsea et al., 2021
CRS757	N/A	0.62 µM	Sensitive	Chronic rhinosinusitis	Onsea et al., 2021
CRS612	N/A	1.25 µM	Sensitive	Chronic rhinosinusitis	Onsea et al., 2021
SA9	N/A	0.06 µM	Sensitive	Bovine mastitis	García et al., 2007
HS121	N/A	0.31 µM	Sensitive	Hidradenitis Suppurativa	Bens et al., 2024
HS035	N/A	1.25 µM	Sensitive	Hidradenitis Suppurativa	Bens et al., 2023
HS120	N/A	0.31 µM	Sensitive	Hidradenitis Suppurativa	Bens et al., 2024
HS009	N/A	0.31 µM	Sensitive	Hidradenitis Suppurativa	Bens et al., 2024
HS008	N/A	0.25 µM	Sensitive	Hidradenitis Suppurativa	Bens et al., 2024
HS131	N/A	1.25 µM	Sensitive	Hidradenitis Suppurativa	Bens et al., 2023
IT-MSSA53	N/A	0.25 µM	Sensitive	Central venous Catheter	Ponce Benavente et al., 2024
CRS613	N/A	0.15 µM	Sensitive	Chronic rhinosinusitis	Onsea et al., 2021
