# Supplementary material for: FibriLysins disrupt Staphylococcus aureus biofilms and abscess communities
Source: Nat Microbiol. 2026 Oct 23;11(11):3133–43. doi: 10.1038/s41564-026-02513-1 (PMC13739733; doi:10.1038/s41564-026-02513-1)

Figure 1C Left

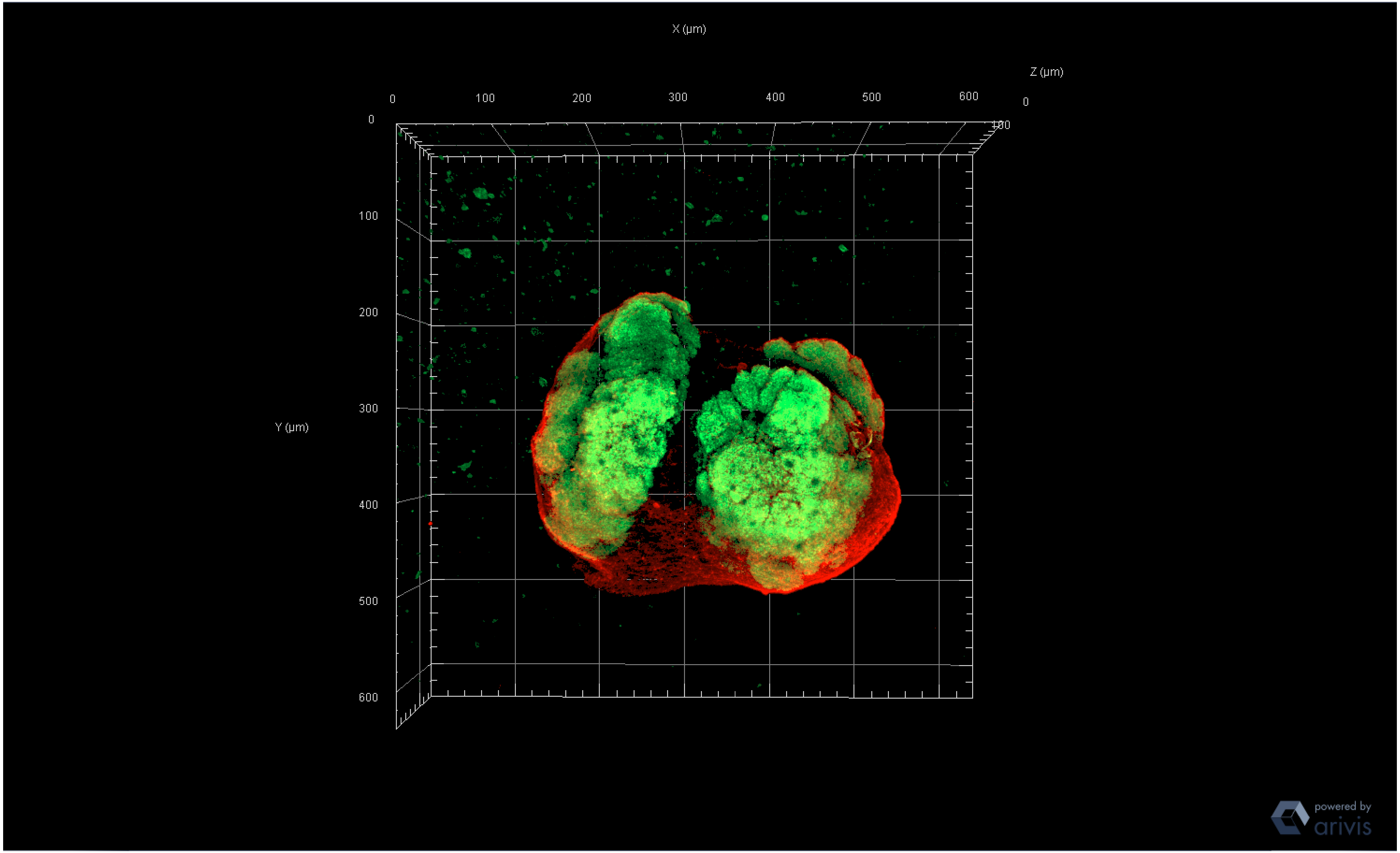

Figure 1C (center)

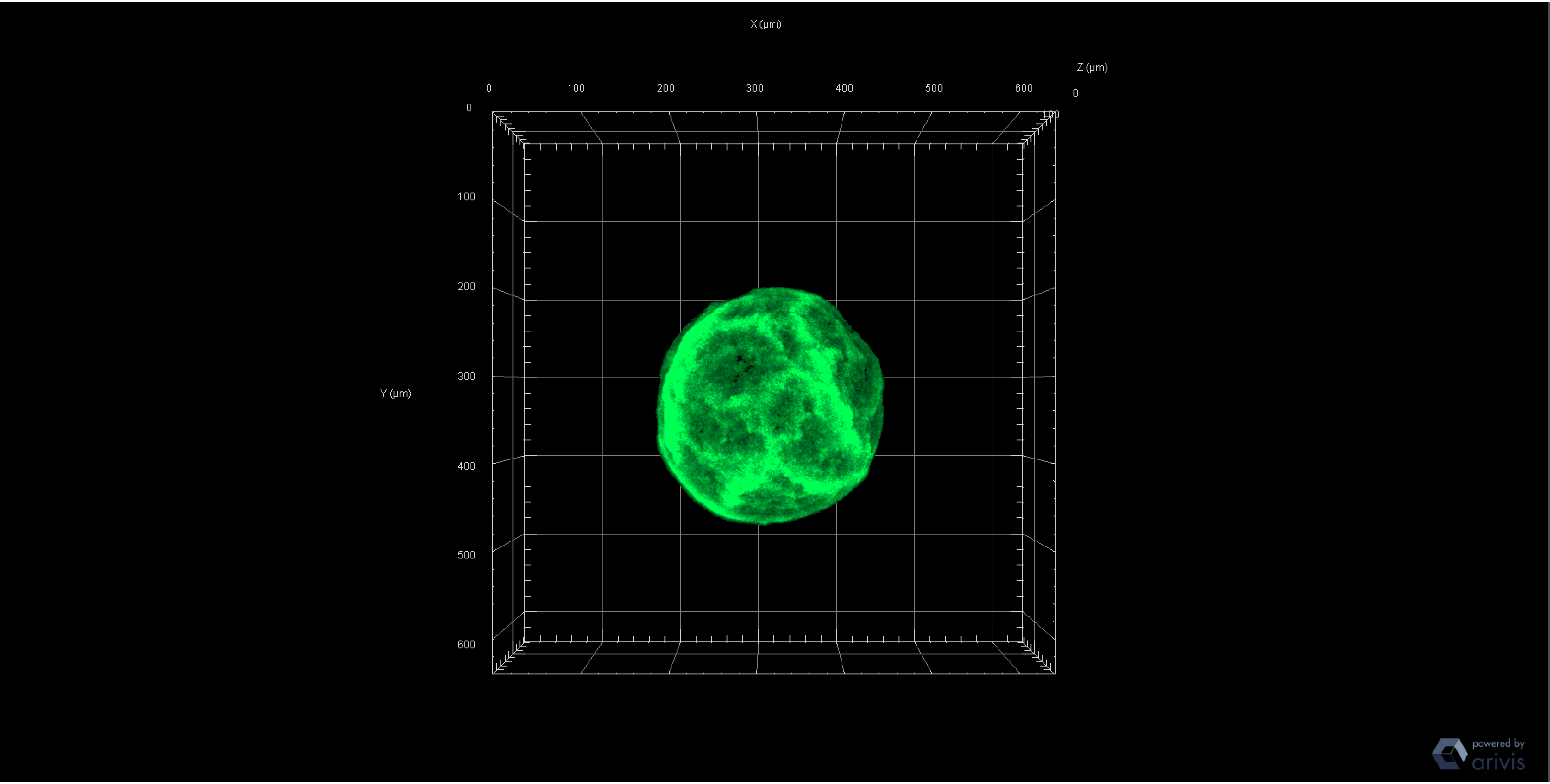

Figure 1C (right)

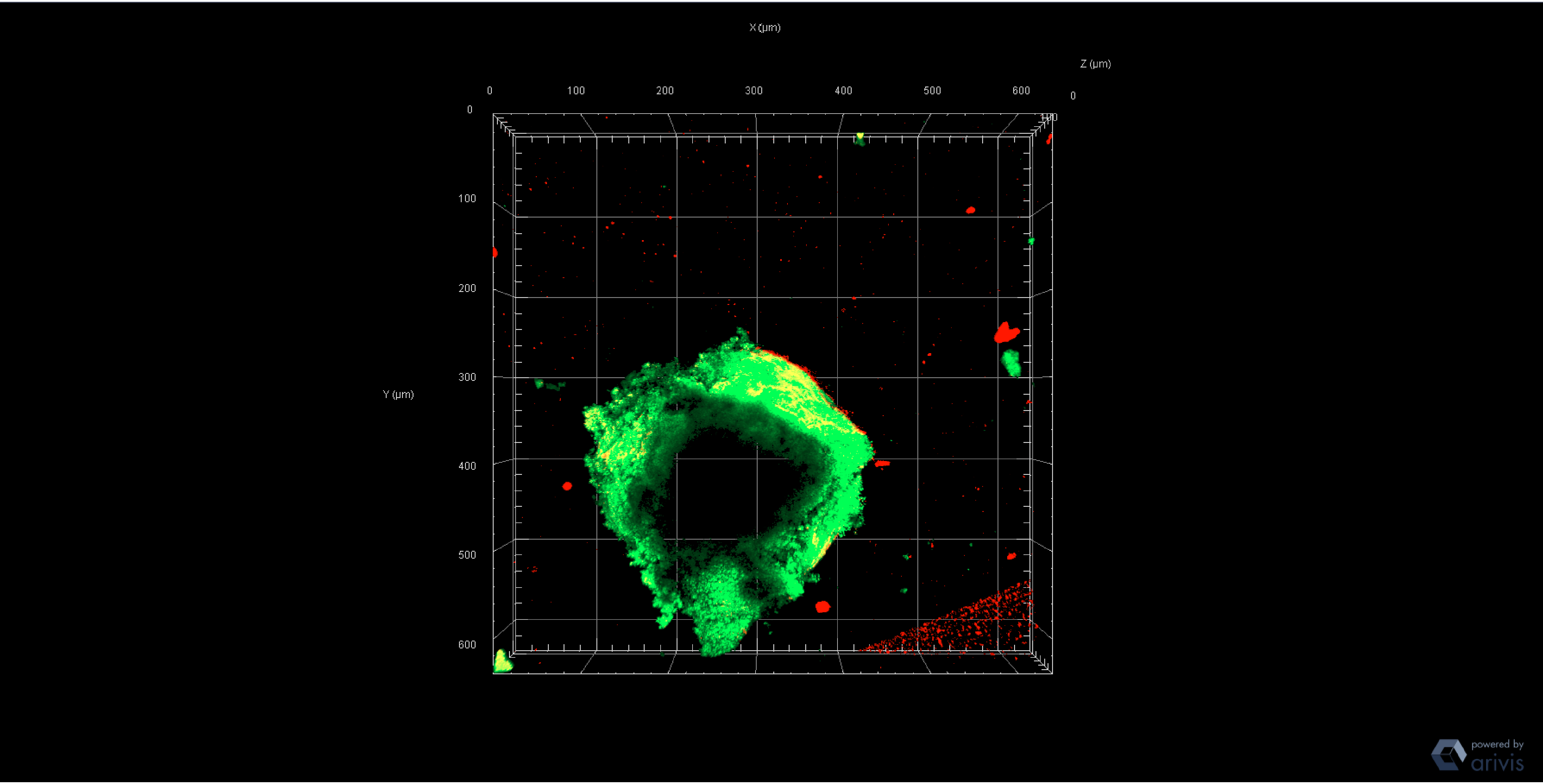

Supplement: Supplementary file 5 [file 41564_2026_2513_MOESM3_ESM.pdf]
